# Development of domain-specific probes of *Plasmodium falciparum* heat shock protein 70-1

**DOI:** 10.1128/aac.00466-26

**Published:** 2026-07-02

**Authors:** Aaron M. Keeler, Gaini Ibrasheva, Elizabeth G. Boger, Yueqi Chen, Porter E. Petruzziello, Erin A. Schroeder, Christopher R. Mansfield, Kayla Sylvester, Cameron D. Fleming, Philip F. Hughes, Timothy A. J. Haystead, Michael C. Fitzgerald, Emily R. Derbyshire

**Affiliations:** 1 Department of Chemistry, Duke University 201942, Durham, North Carolina, USA; 2 Department of Molecular Genetics and Microbiology, Duke University Medical Center 609772, Durham, North Carolina, USA; 3 Department of Pharmacology and Cancer Biology, Duke University Medical Center 609772, Durham, North Carolina, USA; The Children's Hospital of Philadelphia, Philadelphia, Pennsylvania, USA

**Keywords:** domain-specific, heat shock protein, Hsp70, malaria, *Plasmodium*

## Abstract

In the malaria parasite *Plasmodium falciparum*, the essential chaperone *Pf*Hsp70-1 regulates proteostasis through protein folding, but its domain-specific functions remain poorly defined. The protein contains an N-terminal nucleotide-binding domain (NBD) and a C-terminal substrate-binding domain (SBD), where selective inhibitors are needed to probe domain-specific functions and advance *Pf*Hsp70-1 as an antimalarial drug target. Here, we identified small molecules that bind *Pf*Hsp70-1 using a high-throughput thermal shift screen, an unbiased approach that enables the discovery of ligands targeting any *Pf*Hsp70-1 domain. Molecules were prioritized by their affinity to *Pf*Hsp70-1 and anti-*Plasmodium* activity. These efforts led to the characterization of AMK3 and AMK4, which bind to *Pf*Hsp70-1 with low-micromolar affinity and exhibit selectivity (>25-fold) for *Pf*Hsp70-1 over the human homolog HSPA1A based on microscale thermophoresis. The *Pf*Hsp70-1 binding regions of AMK3 and AMK4 were isolated using a combination of proteomic and biochemical methods, which demonstrated that AMK3 binds to the NBD and competes with ATP binding. In contrast, AMK4 binds to the SBD and leads to disruption in peptide binding. A molecular dynamic-facilitated structure-activity relationship (SAR) screen was performed on AMK3, yielding a compound with retained anti-*Plasmodium* activity but reduced host cytotoxicity. This study identifies species-selective N-terminal and C-terminal inhibitors of *Pf*Hsp70-1, and suggests druggable binding pockets on the C-terminal domain that may be exploited for the disruption of essential protein-protein interactions. These domain-specific inhibitors are useful starting points for the development of probes to advance our knowledge of *Pf*Hsp70-1 functions during infection and as therapeutics to treat malaria.

## INTRODUCTION

Malaria is a widespread global health burden that is responsible for over 600,000 deaths each year, primarily in sub-Saharan Africa and Southeast Asia (1). The causative agent of malaria is the apicomplexan *Plasmodium* parasite, where a single species, *P. falciparum*, is responsible for nearly all human malarial deaths. *Plasmodium* undergoes a complex life cycle traversing from a cold-blooded female *Anopheles* mosquito to a warm-blooded host where it first infects hepatocytes prior to the infection of red blood cells. The cyclical infection and destruction of red blood cells drive malaria symptoms, including periodic fevers (2). During its life cycle, *Plasmodium* endures a significant number of cellular stresses, particularly heat stress. To successfully develop and replicate in these distinct microenvironments, *Plasmodium* relies on heat shock proteins (Hsps) to maintain homeostasis (3, 4).

Hsps are ubiquitous chaperone proteins that facilitate cellular processes throughout all kingdoms. Hsps are known to support protein folding, stabilization, and maturation, which globally suppresses protein aggregation and maintains proteostasis (5–8). Within this family, heat shock protein 70 (Hsp70) isoforms are highly conserved chaperones that have been well-studied in yeast, bacteria, and humans due to their essential role in the stress response. These studies have demonstrated that Hsp70s consist of an N-terminal nucleotide-binding domain (NBD) capable of ATP hydrolysis, and a C-terminal region containing the substrate-binding domain (SBD) and a disordered region that often includes an EEVD tail sequence (9, 10). The SBD is further divided into two subdomains, a β-sheet subdomain (SBDβ) that contains the peptide binding site, and an α-helical subdomain (SBDα), also referred to as the lid domain. The SBD is known to interact with other chaperones and co-chaperones, including Hsp90 and heat shock organizing protein. Similarly, the NBD has also been shown to interact with Hsp40, Hsp90, and nucleotide exchange factors, to maintain proteostasis (11–14). Thus, both the NBD and SBD have critical yet distinct functions where the interplay between these domains is crucial for supporting Hsp70 function (7, 15–17).

In *P. falciparum*, *Pf*Hsp70-1 (UniProt access: Q8IB24) is the most extensively characterized isoform. This protein predominantly functions in the cytoplasm, but also translocates to the nucleus and is highly upregulated during stress conditions (8, 18). Importantly, the protein is essential throughout the parasite’s life cycle, making it an attractive drug target (8, 19, 20). To investigate the functional role of Hsp70-1, we recently employed the non-selective Hsp70-1 C-terminal EEVD inhibitor 15-deoxyspergualin. This approach revealed its heat stress-associated role in maintaining digestive vacuole stability, an essential lysosome-like organelle unique to *Plasmodium* that supports hemoglobin digestion (21). While this highlighted the protein’s unique role in *Plasmodium*, further studies were hindered due to the current deficiency in species-selective chemical probes.

Hsp70-1-binding molecules have been extensively pursued because of the protein’s important role across a wide range of diseases. Significant efforts have been dedicated to the identification and development of N-terminal, C-terminal, and allosteric small molecule modulators, particularly for human Hsp70-1 isoforms, with considerable success (22–27). Similar approaches have been explored in *Plasmodium* using both recombinant proteins and *in vitro* parasite screening. For example, members of the pyrimidinones class (DMT2264, MAL3-39) were found to inhibit ATPase activity at high concentrations, whereas malonganenoes A-C, lapachol, and bromo-β-lapachona inhibited the aggregation suppression activity of *Pf*Hsp70-1 without impacting ATPase activity (19, 28, 29). The polyphenol epigallocatechin 3-gallate (EGCG) and the lipopeptide polymyxin B were identified as probable N-terminal binders that inhibit the basal ATPase activity of *Pf*Hsp70-1 and its association with co-chaperones *in vitro* while suppressing parasite growth (30, 31). More recently, iso-mukaadial acetate (IMA) and ursolic acid (UAA) were found to inhibit the aggregation suppression activity of *Pf*Hsp70-1 in a concentration-dependent manner (32). Both molecules exhibit anti-parasitic activity, but only IMA, not UAA, inhibits *Pf*Hsp70-1 basal ATPase activity. These molecules provide valuable starting points for *Pf*Hsp70-1 ligands, but concerns for non-specific binding, off-target effects, and poor bioavailability have all challenged further development. Specifically, *Plasmodium*-selective C-terminal and N-terminal Hsp70-1-binding molecules would be particularly valuable; however, the high sequence similarity (73%) between the parasite and human proteins (HSPA1A UniProt access: P0DMV8), especially in the ATP-binding site (77%), has hindered rational drug design (33).

Herein, we employed a high-throughput target-based thermal stability screen to identify small molecules that bind to full-length *Pf*Hsp70-1. From this screen, four compounds named AMK1-4 were prioritized based on the novelty of their chemical scaffolds, affinity to *Pf*Hsp70-1, and anti-*Plasmodium* activity. Each compound was further characterized for *Pf*Hsp70-1 ATPase inhibition. These studies indicated AMK3 and AMK4 were >25-fold selective for *Pf*Hsp70-1 binding over HSPA1A, the greatest species selectivity reported to date. To better understand the basis for this selectivity, biochemical and proteomic strategies were used to inform the small-molecule binding sites. We demonstrate that AMK3 binds to the *Pf*Hsp70-1 N-terminal NBD and competes for ATP binding, while AMK4 targets a site on the C-terminus. While both AMK3 and AMK4 inhibit ATPase activity, only AMK4 disrupted *Pf*Hsp70-1 peptide binding *in vitro*. Collectively, these findings highlight chemical scaffolds with species selectivity and domain-specific binding. These small molecules serve as much-needed starting points for probes to interrogate *Pf*Hsp70-1 function during *Plasmodium* infection and lead to future antimalarial drug development.

## RESULTS

### Thermal shift screen identifies *Pf*Hsp70-1-binding molecules

A high-throughput screen leveraging a thermal shift assay (TSA) was developed to identify compounds that bind to *Pf*Hsp70-1. After purification of full-length recombinant *Pf*Hsp70-1, ATPase activity was assessed to ensure a functional NBD. These experiments established that our determined *K*_M_ and V_max_ for ATP hydrolysis were comparable to published values (Fig. S1A through C) (34, 35). TSA-based screens have been widely employed in recent years due to their efficiency and affordability. Here, we sought to establish the first TSA-based high-throughput screen for *Pf*Hsp70-1 by optimizing protein and positive control concentrations in a 96-well plate format. The Tm of *Pf*Hsp70-1 was assessed at varying protein concentrations and after addition of the positive controls ATP and the previously reported non-selective NBD inhibitor EGCG (21) (Fig. S1D) were assessed (Fig. S2A and B). ATP and EGCG elicited shifts of 3°C and 5°C, respectively, characteristic of a binding interaction (Fig. S2C). A Z′ factor of 0.68 was achieved using 3 µM *Pf*Hsp70-1 with DMSO as the negative control and 1 mM ATP as the positive control, demonstrating a robust screen.

**Fig 1:**
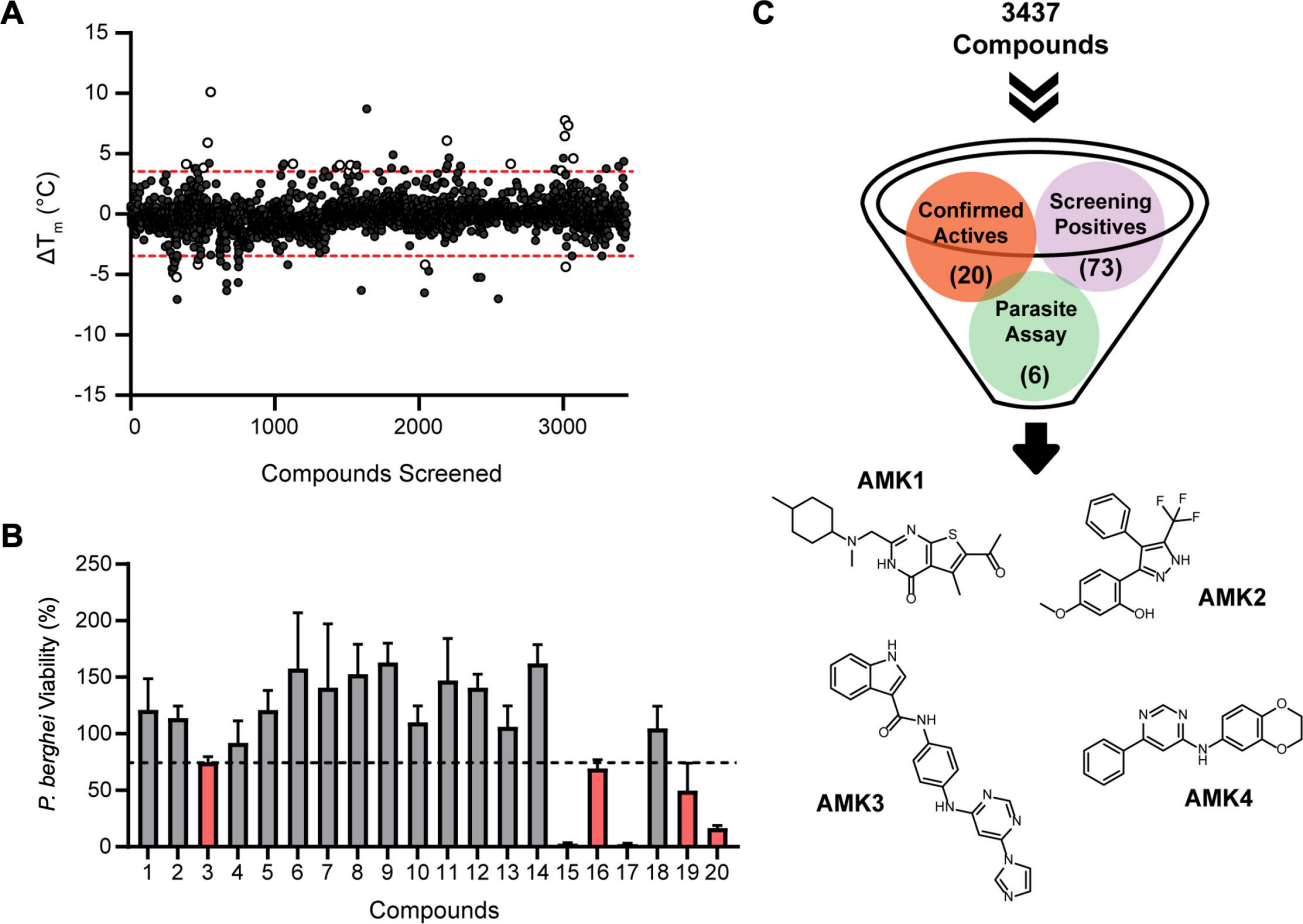
High-throughput screen identifies molecules that bind to *Pf*Hsp70-1. (**A**) Change in melting temperature (Tm) of *Pf*Hsp70-1 with the addition of 3,437 compounds relative to DMSO. Threshold for actives was a Tm change of ≥3.5°C (red dashed line). Actives were confirmed through a 5-point dose-response curve (open circles). (**B**) Confirmed actives (compounds 1–20 at 10 µM) were tested for inhibition of *P. berghei* liver stage parasites, identifying six compounds (red bars) that reduced parasite load (black dashed line denotes 25% inhibition). Data shown as the average ± SEM (*n* = 3). (**C**) Summary of the TSA screening strategy that identified four hits (AMK1–4).

A 3,437-member nucleotide-like library was next assessed at 30 µM to identify molecules that alter the *Pf*Hsp70-1 Tm. Protein-to-compound concentrations were at a 1:10 ratio to allow robust Tm shifts, as has been previously employed (36). From this, 73 screening positives were identified that shifted the *Pf*Hsp70-1 Tm by 3.5°C. This threshold was selected to obtain positives that elicited shifts similar to the ATP control (average Tm shift = 3.5°C and an initial rate of <2.5%. With this cut-off, 39 compounds were identified that increased the Tm ≥ 3.5°C (stabilized) while 34 decreased the Tm ≤ −3.5°C (destabilized), relative to DMSO (Fig. 1A). Screening positives were then tested in the TSA with a 5-point dose-response curve, identifying 20 compounds for further assessment (i.e., dose-response observed). These screening activities were next tested for anti-*Plasmodium* activity with the commonly used *P. berghei* rodent malaria liver stage model, as well as for hepatocyte cytotoxicity (37). Specifically, human hepatoma Huh7 cells are infected with luciferase-expressing parasites to assess parasite viability via luminescence. As *Pf*Hsp70-1 is 94% identical to *P. berghei* Hsp70-1 (*Pb*Hsp70-1), we expect compounds to bind to both proteins (Fig. S3). In addition to identifying molecules with anti-parasite activity, this approach also eliminates cytotoxic compounds that decrease host cell viability. Six compounds (3, 15–17, 19, 20) at 10 µM decreased *P. berghei* ANKA viability >25% without significant cytotoxicity against Huh7 cells (<50%) compared to DMSO (Fig. 1B; Fig. S4A). Among these six compounds, 16 and 19 (HSX-0138) contain imidazole pyrimidine scaffolds, while 17 and 20 (HSX-3193) represent 1,4-benzodioxane scaffolds (Fig. S4B and C). The identification of these structural analogs increased our confidence in the selection of these molecules for further study, where 16 and 17 were prioritized due to their anti-*Plasmodium* activity and commercial availability. Compounds 3, 15, 16, and 17 were then purchased and confirmed to bind *Pf*Hsp70-1 using the TSA. These prioritized compounds are henceforth referred to as AMK1, AMK2, AMK3, and AMK4, respectively (Fig. 1C).

### AMK3 and AMK4 exhibit species-selective binding

**Fig 2:**
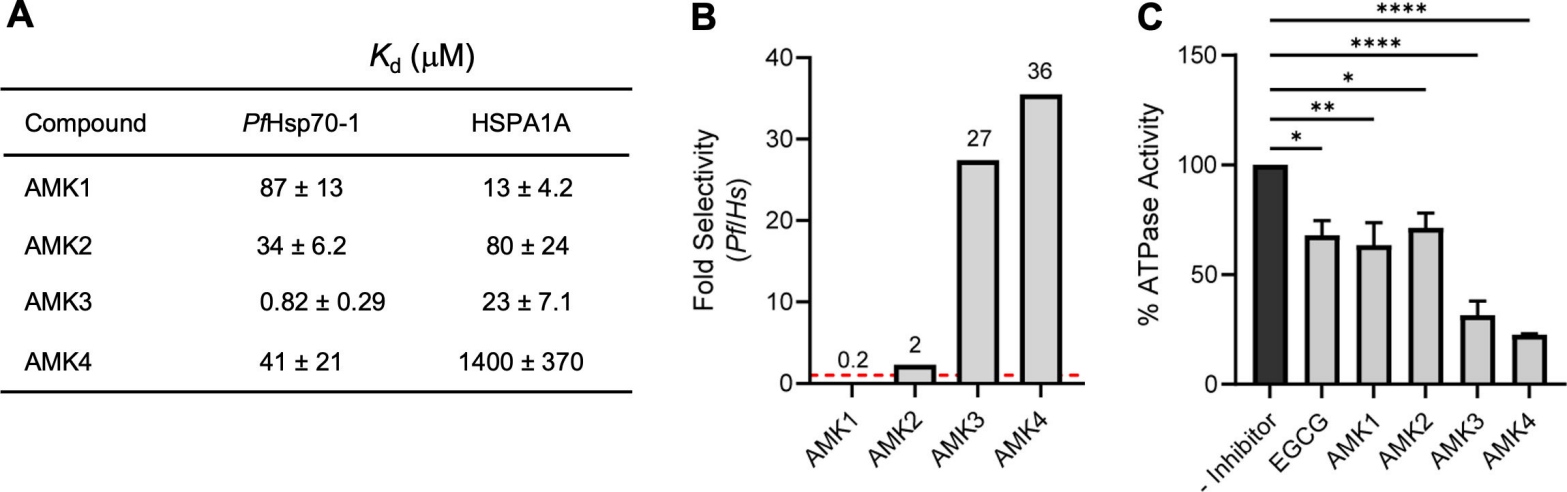
Prioritized compounds exhibit species selectivity and inhibit *Pf*Hsp70-1 ATPase activity. (**A**) Binding affinity of AMK1–4 to *Pf*Hsp70-1 and HSPA1A determined by MST. Data shown as average ± SEM (*n* ≥ 3). (**B**) AMK1–4 fold-selectivity for *Pf*Hsp70-1 over HSPA1A, with the red dashed line denoting onefold (i.e., no selectivity). (**C**) AMK1–4 (30 µM) were tested for inhibition of basal *Pf*Hsp70-1 ATPase activity, with EGCG as a positive control. Data shown as average ± SEM (*n* ≥ 3). *P < 0.05; **P < 0.01; ***P < 0.001 (unpaired t-test) relative to DMSO control.

As an independent means to assess AMK1-AMK4 binding to *Pf*Hsp70-1, as well as potential species selectivity, microscale thermophoresis (MST) was employed (38). First, RED-NHS-labeled purified *Pf*Hsp70-1 and its human ortholog HSPA1A were generated, and the *K*_d_ of EGCG to *Pf*Hsp70-1 was measured. The determined *K*_d_ value of 0.13 ± 0.03 µM is comparable to that previously determined with SPR (0.44 ± 0.07 µM) (31). AMK1-4 binding to labeled *Pf*Hsp70-1 and HSPA1A (Fig. S5) was then assessed, where representative MST binding curves (Fig. S6) and *K*_d_ determinations (Fig. 2A) are shown. From these experiments, AMK1 and AMK2 bound to *Pf*Hsp70-1 but did not exhibit species selectivity toward *Plasmodium* (Fig. 2B), showing fold-selectivities of 0.2 (AMK1) and 2 (AMK2). AMK3 exhibited the highest affinity for *Pf*Hsp70-1 and was species selective (fold-selectivity of 27) with the *Pf*Hsp70-1 *K*_d_ of 0.82 ± 0.29 µM and the HSPA1A *K*_d_ of 23 ± 7.1 µM. AMK4 had a mid-micromolar *K*_d_ of 41 ± 21 µM to *Pf*Hsp70-1 but was the most species selective (fold-selectivity of 36).

### Prioritized compounds inhibit *Pf*Hsp70-1 ATPase activity

Prioritized compounds were also tested for their ability to disrupt ATP hydrolysis of *Pf*Hsp70-1. All compounds at 30 µM significantly decreased *Pf*Hsp70-1 ATPase activity, but AMK3 and AMK4 were the most potent, inducing inhibition greater than the EGCG positive control (Fig. 2C). Of note, the ATPase assay was an endpoint measurement, where the observed reduction in hydrolysis could stem from a change in *K*_M_ and/or V_max_. Due to their potency of ATPase inhibition and species-selective binding, AMK3 and AMK4 were prioritized for subsequent studies.

### AMK3 and AMK4 bind to distinct regions

**Fig 3:**
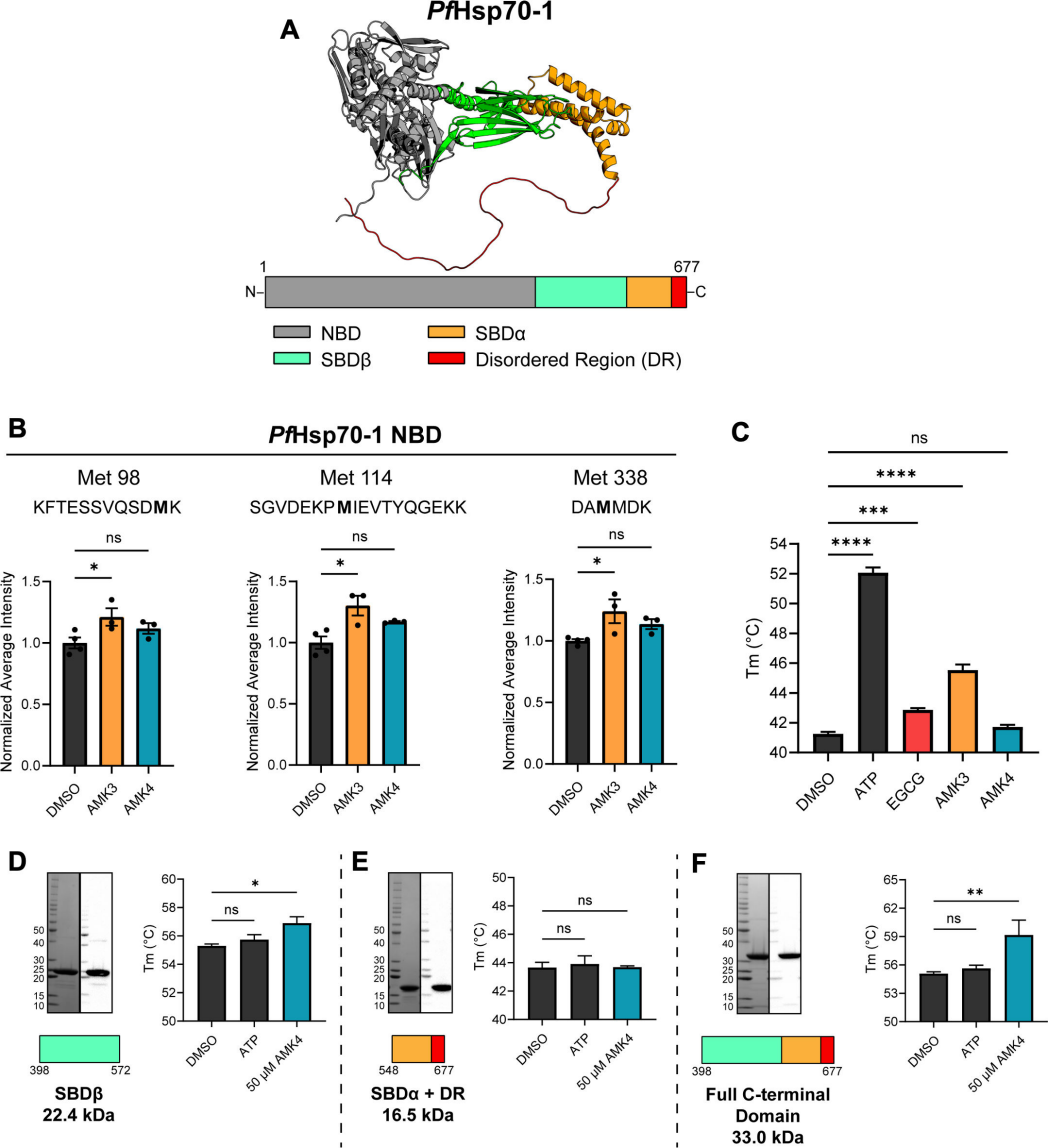
AMK3 and AMK4 bind to distinct locations on *Pf*Hsp70-1. (**A**) *Pf*Hsp70-1 model (Alphafold2) colored by domain: nucleotide binding domain (NBD; grey), substrate binding domain β (SBDβ; green), substrate binding domain α (SBDα; orange), and disordered region (DR; dark red) indicated. (**B**) SPROX analysis of *Pf*Hsp70-1 methionine-containing peptides that map to the *Pf*Hsp70-1 NBD (M98, M114, and M338) demonstrates stabilization with 250 µM AMK3 (orange) but not AMK4 (blue) relative to DMSO (dark grey). Analysis was performed using *E. coli* lysate with *Pf*Hsp70-1 overexpressed. Data shown as average ± SEM (*n* = 3–4). *P < 0.05; ns: not significant (one-way ANOVA). (**C**) Tm analysis for *Pf*Hsp70-1 NBD. Tm shifts were completed with *Pf*Hsp70-1 NBD (3 µM) with 1 mM ATP or 200 µM EGCG, AMK3, or AMK4, which indicates ATP, EGCG, and AMK3 bind to the NBD. Data shown as average ± SEM (*n* = 5). SDS-PAGE gels, western blots, and Tm analyses for *Pf*Hsp70-1 SBDβ (**D**), SBDα+DR (**E**), and full C-terminal (**F**) domains. Tm shifts were completed with DMSO, ATP (1 mM), and AMK4 (50 µM) and demonstrate AMK4 binds to the SBDβ and full C-terminal domains. Data shown as average ± SEM (*n* ≥ 3). (**C–F**) *P < 0.05; **P < 0.01; ***P < 0.001; ****P < 0.0001; ns, not significant (unpaired t-test).

The bottom-up proteomics methods, stability of proteins from rates of oxidation (SPROX) and pulse proteolysis (PP), were employed to probe the AMK3 and AMK4 *Pf*Hsp70-1 binding site(s) (Fig. 3A). SPROX, which maps differences in modified methionines after H_2_O_2_ oxidation, was performed with *Pf*Hsp70-1 overexpressed in *Escherichia coli* with and without AMK3 or AMK4 (250 µM) incubation. The negative control was DMSO (5%). Several methionine-containing peptides that mapped to the NBD of *Pf*Hsp70-1 were observed, and these peptides showed increased stability in the presence of AMK3 when compared to DMSO. In contrast, there was no apparent change in the stability of these NBD peptides in the presence of AMK4 (Fig. 3B). Unfortunately, there was less peptide coverage of the *Pf*Hsp70-1 C-terminal domain due to the absence of methionine residues. Only one methionine-containing peptide mapped to the *Pf*Hsp70-1 SBDβ domain, and this showed no change in stability with AMK3 or AMK4 compared to the DMSO control (Fig. S7A). It is unknown if changes in peptide stability were not observed with AMK4 due to its relatively high *K*_d_ (~50-fold higher than AMK3) or if coverage was lacking for the relevant binding site; however, these data support the binding of AMK3 to the *Pf*Hsp70-1 N-terminal domain.

To further assess the AMK3 and AMK4 binding site(s), a semitryptic peptide enrichment strategy for proteolysis procedures (STEPP) coupled to PP (STEPP-PP) was performed on lysate after *Pf*Hsp70-1 overexpression in *E. coli*. This method does not rely on the presence of methionine residues and provides complementary information to SPROX on protein stability. *Pf*Hsp70-1 lysate was incubated with and without AMK3 or AMK4 (250 µM). All samples and controls had a final DMSO concentration of 5%. With AMK3, a statistically significant decrease in a semitryptic peptide that mapped to the *Pf*Hsp70-1 NBD was observed, while there was an increase in intensity for multiple semitryptic peptides that mapped to the SBDβ (Fig. S7B). In contrast, no significant change for any semitryptic peptide was observed with AMK4 relative to the DMSO control. Again, there was less coverage of the *Pf*Hsp70-1 C-terminal domain when compared to the NBD, which may have obscured detection of AMK4 binding (Fig. S7C).

We next sought to obtain biochemical support for the AMK3 and AMK4 binding sites by isolating various *Pf*Hsp70-1 truncations. First, the known NBD truncation *Pf*Hsp70-1 NBD (1–398) was cloned, expressed, and purified to homogeneity (Fig. S5C). To assess secondary structure, the circular dichroism (CD) spectrum of *Pf*Hsp70-1 NBD (1–398) was compared to full-length *Pf*Hsp70-1, which supports their predicted structures (Fig. S8A and B). The ability of the protein to bind to ATP was validated using the TSA (Fig. S5D) (39). The assay was then used to test if AMK3 or AMK4 binds to *Pf*Hsp70-1 NBD (3 µM), with ATP and EGCG employed as positive controls (Fig. 3C). In these assays, an excess of ligand (200 µM) was used to detect any possible binding event. AMK3, ATP, and EGCG induced a significant shift in the *Pf*Hsp70-1 NBD Tm, while AMK4 showed no significant shift compared to the DMSO control. These data suggest that AMK3 but not AMK4 binds to the *Pf*Hsp70-1 N-terminal domain.

With our proteomic and biochemical data suggesting that AMK4 may bind outside of the *Pf*Hsp70-1 NBD, several *Pf*Hsp70-1 C-terminal domain truncations were designed using previously determined domain boundaries and guidance with Alphafold2 structure predictions (40). Specifically, *Pf*Hsp70-1 SBDβ (398-572), *Pf*Hsp70-1 SBDα+disordered region (DR) (548–677), and the entire C-terminal domain (398–677) were cloned, expressed, and purified. These constructs were evaluated by CD spectroscopy to assess secondary structure and protein folding, where spectra were consistent with predicted homology models (Fig. S8C through E). Each truncation was then assessed for AMK4 (50 µM) binding using a TSA (Fig. 3D through F). This concentration was selected based on the determined AMK4 *K*_d_ of 40.6 µM to full-length *Pf*Hsp70-1. In these studies, ATP (1 mM) was used as a negative control. As expected, ATP did not induce a Tm shift in any of the C-terminal *Pf*Hsp70-1 truncations. In contrast, AMK4 induced a Tm shift in *Pf*Hsp70-1 SBDβ and the full C-terminal domain, but not the *Pf*Hsp70-1 SBDα+DR truncation. While the observed *Pf*Hsp70-1 SBDα+DR Tm and CD spectrum support secondary structure, no known ligand exists for this domain to serve as a positive control in the binding assay. Consequently, the absence of detectable AMK4 binding in the TSA may reflect partial protein misfolding (i.e., false negative). However, these data directly support the proposal that AMK4 binds to the SBDβ of *Pf*Hsp70-1.

The DeepSite webserver was next used to identify putative pockets on the *Pf*Hsp70-1 SBDβ domain (398–572) based on an Alphafold2 model (41, 42). AMK4 was docked to each putative site (AutoDock Vina), and protein-ligand binding was visualized using the Protein-Ligand Interaction Profiler (PLIP) webserver, which enables prediction of non-covalent interactions (Fig. S9) (43–45). From this computational analysis, three predicted potential binding pockets within the SBDβ domain were identified, with site 1 (the known site of peptide binding) having the largest volume and the most favorable predicted binding energy (−8.6 kcal/mol). Site 2 was the least favored by docking energy (−5.9 kcal/mol) when compared to sites 1 and 3 (−7.8 kcal/mol).

### AMK4 disrupts peptide binding to *Pf*Hsp70-1

**Fig 4:**
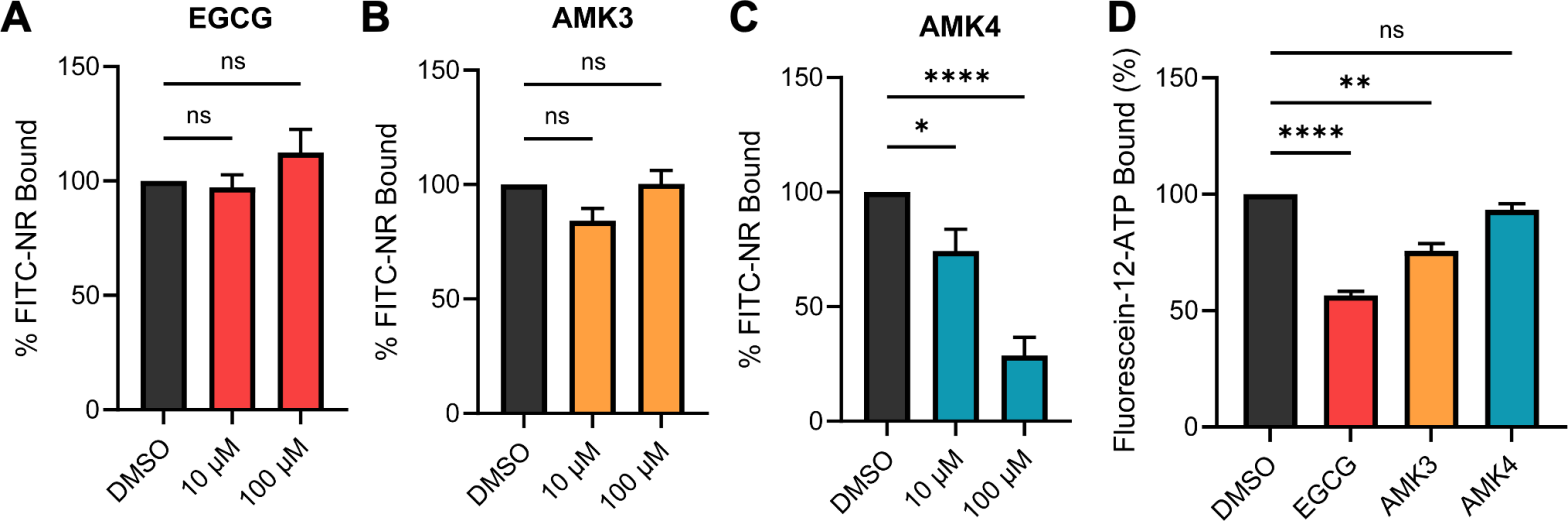
AMK3 and AMK4 differentially affect *Pf*Hsp70-1 peptide and ATP binding. FITC-NR peptide (10 nM) binding to *Pf*Hsp70-1 (1.3 µM) in the presence of EGCG (**A**), AMK3 (**B**), or AMK4 (**C**). (**D**) F12-ATP (5 nM) binding to *Pf*Hsp70-1 NBD (100 nM) in the presence of EGCG, AMK3, and AMK4 (100 µM). (**A–D**) Data shown as average ± SEM (*n* = 3–5). *P < 0.05; **P < 0.01; ****P < 0.0001; ns, not significant (unpaired t-test) relative to DMSO control.

The ability of AMK3 or AMK4 to influence peptide binding to *Pf*Hsp70-1 was assessed using an established NR (NRLLLTG) peptide fluorescence polarization assay (46, 47). Using synthesized FITC-coupled NR peptide (10 nM) and increasing concentrations of full-length *Pf*Hsp70-1, the *K*_d(app)_ of 1.29 ± 0.05 µM was determined (Fig. S10A), which is comparable to *K*_d_’s reported for Hsp70-1 homologs from other organisms (46, 47). FITC-NR binding to the *Pf*Hsp70-1 NBD, SBDβ, SBDα+DR, and full C-terminal domain was also assessed (Fig. S10B). As expected, FITC-NR only binds to full-length *Pf*Hsp70-1 and the SBDβ domain. AMK3, AMK4, and EGCG were then tested at 10 and 100 µM for the ability to disrupt peptide binding to full-length *Pf*Hsp70-1. No significant change in peptide binding was observed with the N-terminal inhibitors EGCG or AMK3 relative to the DMSO control at either concentration (Fig. 4A and B). In contrast, AMK4 significantly decreased FITC-NR binding to *Pf*Hsp70-1 at 100 µM (Fig. 4C).

### AMK3 competes with ATP binding

AMK3 and AMK4 were also tested for their ability to compete with ATP binding at the *Pf*Hsp70-1 NBD using a previously described fluorescent polarization assay that employs fluorescein-12-ATP (F12-ATP) (48). Using F12-ATP (5 nM) and increasing concentrations of purified *Pf*Hsp70-1 NBD, the *K*_d(app)_ of 8.0 ± 0.3 nM of F12-ATP to *Pf*Hsp70-1 was obtained (Fig. S11). When tested at 100 µM, AMK3 and the positive control EGCG significantly decreased the percentage of F12-ATP bound to the *Pf*Hsp70-1 NBD, but not AMK4 (Fig. 4D). These data suggest that AMK3 competes with ATP binding.

**Fig 5:**
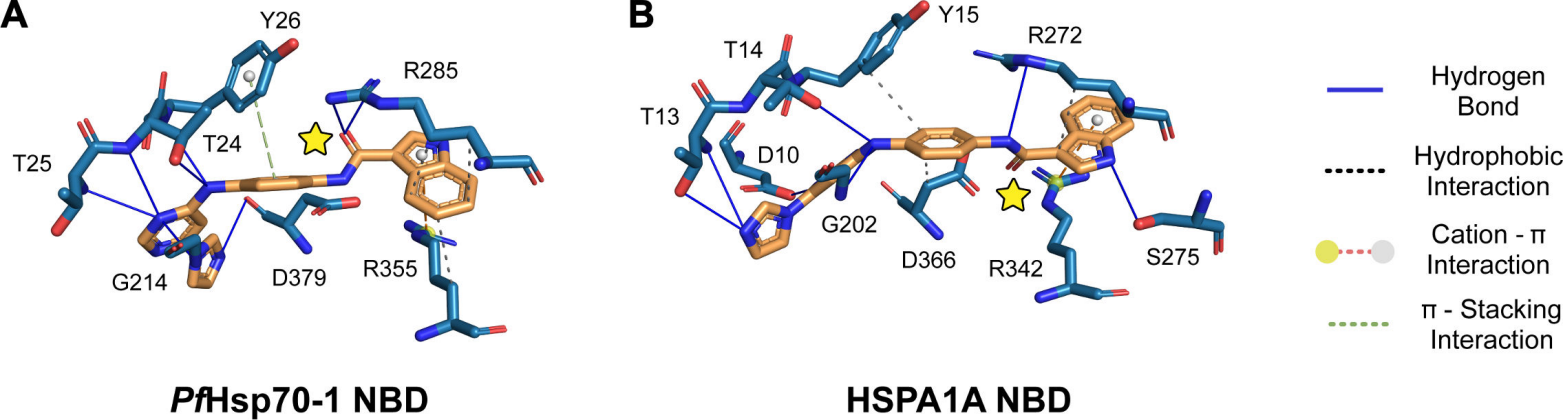
Docking analysis of AMK3. (**A**) Top position of AMK3 docked into *Pf*Hsp70-1 NBD using AutoDock Vina with high exhaustiveness. The PLIP binding interactions of AMK3 (tan) with *Pf*Hsp70-1 NBD residues (blue, sticks) are labeled. The calculated AutoDock Vina docking energy is −9.9 kcal/mol. (**B**) Top position of AMK3 docked into HSPA1A NBD (PDB ID: 3ATV) using AutoDock Vina with high exhaustiveness. The PLIP binding interactions of AMK3 (tan) with HSPA1A NBD residues (blue, sticks) are labeled. The calculated AutoDock Vina docking energy is −11.1 kcal/mol. A predicted hydrogen bonding interaction with the amide bond carbonyl of AMK3 is marked (yellow star).

The binding of AMK3 to the ATP-binding pockets of *Pf*Hsp70-1 (Alphafold2 model) and HSPA1A (PDB ID: 3ATV, crystal structure of ADP-bound state) was investigated with molecular docking and residue interaction analysis. The *Pf*Hsp70-1 NBD (1–398) model was aligned with the structurally similar crystal structure of the *Pf*Hsp70-x NBD (PDB ID: 6S02) to ensure the active site was intact (Fig. S12) (49). Energetically favorable interactions were predicted (AutoDock Vina) with both *Pf*Hsp70-1 (−9.9 kcal/mol) and HSPA1A (−11.1 kcal/mol) (Fig. 5A and B). From these models, differences in AMK3 binding are observed between human versus *P. falciparum* Hsp70-1, including in the orientation of the terminal imidazole ring.

### AMK3 inhibits blood stage *P. falciparum* and binds *Pf*Hsp70-1 in parasite lysate

**Fig 6:**
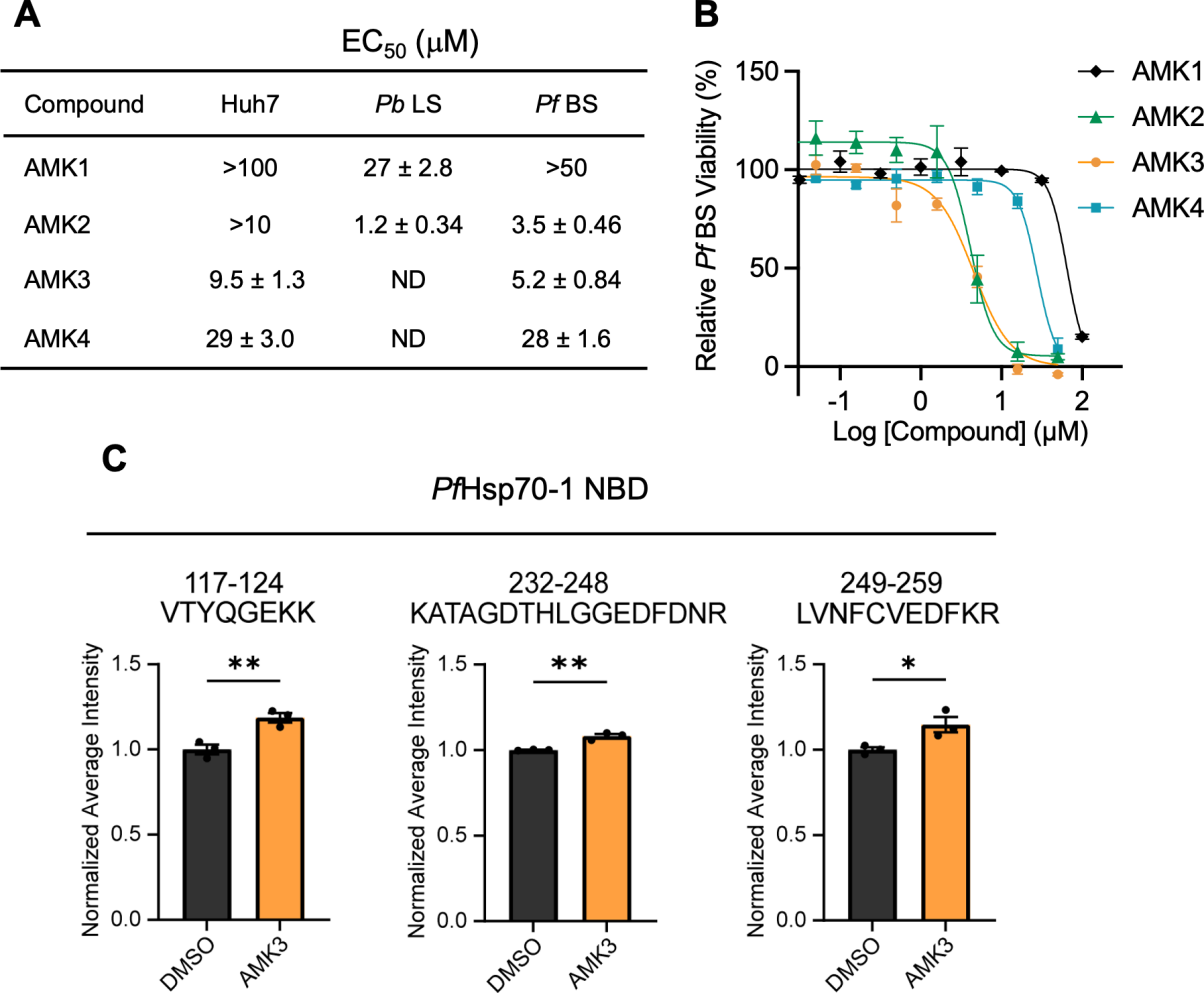
AMK3 inhibits blood stage *P. falciparum* and binds to *Pf*Hsp70-1 in parasite lysate. (**A**) EC50 values of AMK1–4 against Huh7 cells, liver stage *P. berghei* ANKA, and blood stage *P. falciparum* 3D7. Data shown as average (*n* ≥ 3). ND, not determined due to Huh7 toxicity. (**B**) Dose-response curves for AMK1 (black diamond), AMK2 (green triangle), AMK3 (orange circles), and AMK4 (blue squares) inhibition of *P. falciparum* blood stage 3D7 parasites. Data shown as the average ± SEM (*n* = 3–5). (**C**) STEPP-PP analysis of *Pf*Hsp70-1 with 250 µM AMK3 (orange) relative to DMSO (dark grey). Stabilization was observed for several semitryptic peptides that map to the NBD. Analysis was performed using *P. falciparum* lysate. Data shown as average ± SEM (*n* = 3). *P < 0.05; **P < 0.01 (unpaired t-test).

Compounds were next tested for their efficacy against *P. berghei* ANKA liver stage parasites and human-infective *P. falciparum* 3D7 blood stage parasites (Fig. 6A). When tested in a dose-response in a liver stage *P. berghei* high-throughput screen, AMK1 and AMK2 inhibited parasite viability with EC_50_ values of 27 and 1.2 µM, respectively, without significant host cytotoxicity (Huh7 EC_50_ values >100 and >10 µM, respectively). Unfortunately, AMK3 and AMK4 exhibited host cytotoxicity (10–30 µM) that prevented the determination of the liver stage anti-*Plasmodium* EC_50_. Activity against the *P. falciparum* 3D7 blood stage was next assessed using a high-throughput screening platform that quantifies total parasite DNA in erythrocytes 72 h post-treatment. Importantly, erythrocytes are anucleated, allowing the assay to selectively measure parasite DNA. AMK2 and AMK3 were the most potent, exhibiting low micromolar EC_50_ values of 3.5 and 5.2 µM, respectively, while AMK4 (EC_50_ = 28 µM) and AMK1 (EC_50_ > 50 µM) were less potent (Fig. 6B). From this, AMK3 emerged as more compelling than AMK4 for further investigation due to its efficacy against human-infective blood stage parasites.

To confirm cellular on-target binding of AMK3 in *P. falciparum,* STEPP-PP was employed. In this study, *P. falciparum* lysate was treated with AMK3 (250 µM) or a DMSO negative control before STEPP-PP and quantitative MS analysis. From this, several peptides that map to the NBD of *Pf*Hsp70-1 were observed with increased stability in the presence of AMK3 when compared to DMSO, supporting a protein-ligand interaction (Fig. 6C).

### Molecular dynamics-facilitated SAR of AMK3 identifies analog with reduced cytotoxicity

**Fig 7:**
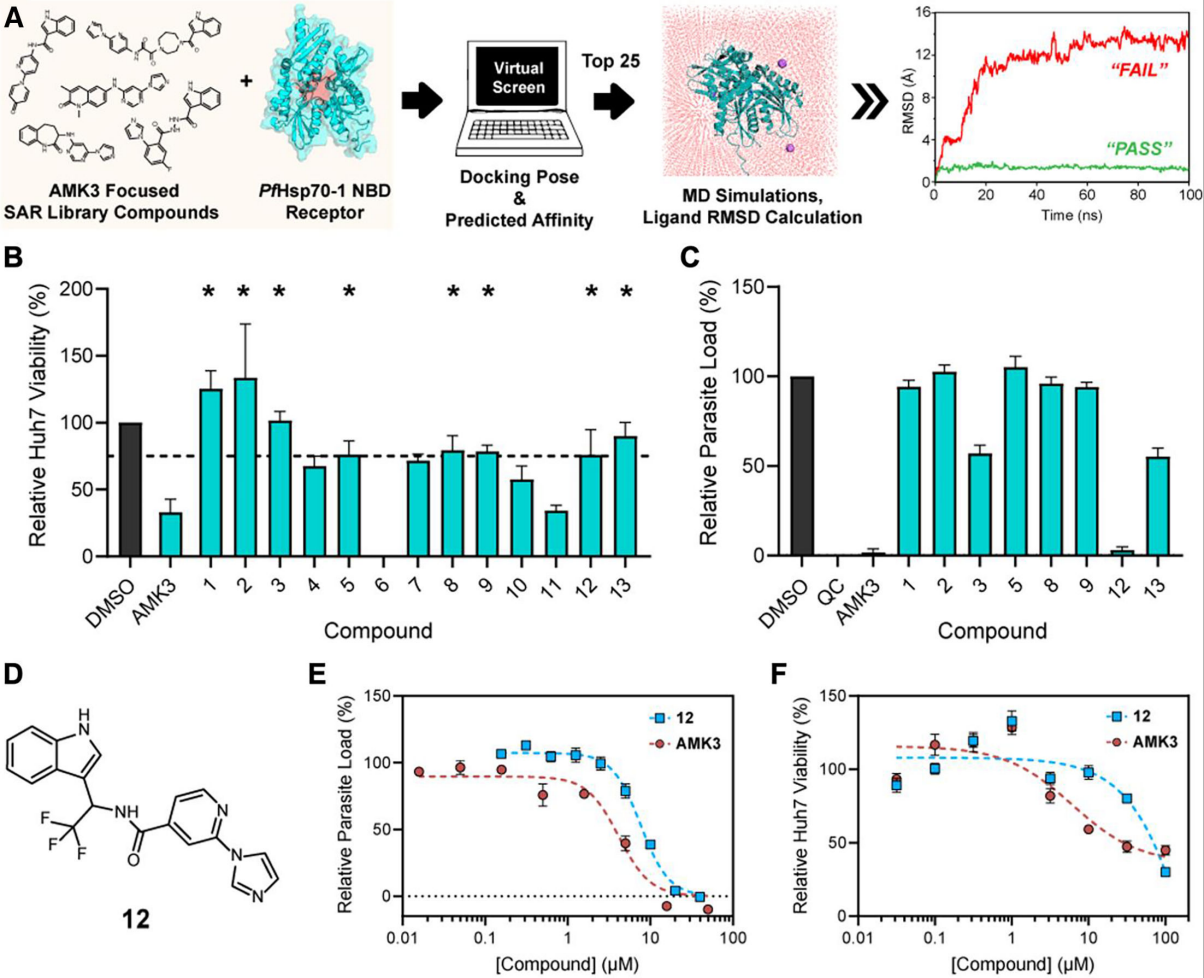
Virtual screen identifies AMK3 analog with reduced hepatocyte cytotoxicity. (**A**) Summary of molecular dynamic-mediated screening strategy. (**B**) Huh7 viability with 13 AMK3 analogs (30 µM). Data normalized to DMSO and shown as average ± SEM (*n* = 5) relative to DMSO control. Prioritized compounds indicated with an asterisk (≥75% viability, above black dotted line). (**C**) *P. falciparum* blood stage parasite viability with eight prioritized compounds (30 µM), negative control DMSO, and positive control quinacrine (QC, 1 µM) indicates compound 12 is the most potent analog. Data shown as average ± SEM (*n* = 4) relative to DMSO control. (**D**) Structure of compound 12. Dose-response curves for compound 12 (blue squares) and AMK3 (red circles) inhibition of *P. falciparum* blood stage parasites (**E**) and Huh7 cells (**F**). Data shown as average ± SEM (*n* = 3–5).

AMK3 exhibits species selectivity to *Pf*Hsp70-1 and displays on-target binding in *P. falciparum* blood stage lysate, but its liver cell cytotoxicity limits its utility as a chemical probe. To address this problem, a molecular dynamic (MD)-assisted structure-activity relationship (SAR) analysis was performed on 200 commercially available compounds that are structurally similar to AMK3 (Enamine). AMK3 competes with ATP binding, so ADP, EGCG, and AMK3 were docked to the *Pf*Hsp70-1 NBD (1-398) model (Alphafold2) using AutoDock Vina and visualized using the PLIP webserver (Fig. S13A through C) (43–45). The interactions predicted for ADP are consistent with expectations based on its binding to structurally similar proteins. A virtual screen of the 200-compound collection was completed by first docking to the *Pf*Hsp70-1 NBD with a high exhaustiveness (AutoDock Vina) and then conducting multiple long time-scale, 100 ns, MD simulations on the top 25 complexes based on docking energy (Fig. 7A). From this, 15 complexes had limited ligand movement, where ligand remained inside the active site throughout the simulations, while 10 failed (Fig. S13D and E) (50, 51). Of these 15 compounds, the 13 that were commercially available (Enamine) were assessed for inhibition of Huh7 cells at 30 µM, identifying 8 that did not decrease cell viability >25% compared to DMSO (Fig. 7B). These eight compounds were tested for activity against blood stage *P. falciparum* at 30 µM (Fig. 7C), where compound 12 (Fig. 7D) decreased *P. falciparum* viability similar to AMK3. Subsequent dose-response studies demonstrated that compound 12 has an EC_50_ of 7.6 µM against *P. falciparum*, similar to AMK3 (Fig. 7F), but significantly improved cytotoxicity (Fig. 7E). Binding to the *Pf*Hsp70-1 NBD was then validated in the TSA (Fig. S14A and B), and MST was used to measure affinity. Compound 12 binds to *Pf*Hsp70-1 with a *K*_d_ of 0.86 ± 0.35 µM and HSPA1A with a *K*_d_ of 3.91 ± 2.66 µM (Fig. S14C), yielding a *Plasmodium* fold-selectivity of 4.5 ± 3.6.

To explore specific moieties that may affect compound 12 binding, it was docked to both the *Pf*Hsp70-1 and HSPA1A NBDs, similar to the study with AMK3. Compound 12 appears to bind in the same location and with a similar binding energy as AMK3 (−9.1 kcal/mol versus −8.7 kcal/mol, respectively). In these ligand-docked structures, the position of the indole ring is again oriented in a flipped position in the *Plasmodium* structure when compared to that of the human homolog (Fig. S15A and B). Additionally, in the compound 12-bound *Pf*Hsp70-1 model, different residues are predicted to participate in hydrogen bonding, such as K82 and E187 (conserved between *Pf*Hsp70-1 and HSPA1A), that are not predicted to be interactors with binding to HSPA1A. If these differences in binding are confirmed, it presents a future opportunity to design species-selective inhibitors with greater potency.

## DISCUSSION

The multi-domain molecular chaperone Hsp70 has critical functions in eukaryotes, including *Plasmodium* parasites. Its activity is driven by ATP hydrolysis at the NBD, but it is also regulated by co-chaperone binding, such as Hsp40 and nucleotide exchange factors (ex, Hsp110) (14, 52, 53). The C-terminus is critical for client binding, with a preference for binding to unfolded hydrophobic regions. Additionally, the C-terminus is proposed to direct Hsp70 subcellular localization for distinct biological functions (54). Within *Plasmodium*, selective small molecule probes are necessary to untangle the essential functional roles of *Pf*Hsp70-1 domains, but thus far, these molecules have been difficult to achieve. The high sequence identity between *Plasmodium* Hsp70-1 and HSPA1A, and the lack of structural information on the middle and C-terminal regions, all hamper these efforts. To address this, we designed a thermal shift high-throughput screen to capture molecules that bind to any region of *Pf*Hsp70-1. Distinct from screens that detect molecules that bind to the ATP-binding site, our screen was designed to be non-regioselective, as ligand binding anywhere on the full-length protein could induce a Tm shift (55–57). Molecules that inhibited liver stage *Plasmodium* parasites were then selected to focus our efforts on scaffolds best suited for future cell-based studies and antimalarial development. Through this effort, we discovered four compounds with novel structures and no previously reported activity toward *Pf*Hsp70-1 or HSPA1A, termed AMK1-4. We observed that compounds AMK2-4 also inhibited blood stage *P. falciparum* with EC_50_ values in the low micromolar range, indicating dual-stage activity, but cytotoxicity of the most potent compound, AMK3, limits its future use. A virtual screen with molecular dynamic simulations enabled optimization of this imidazole pyrimidine scaffold to yield compound 12, an analog with decreased cellular toxicity but retained anti-*Plasmodium* activity. Future optimization of this scaffold may provide a potent and selective molecule to probe *Pf*Hsp70-1 function throughout infection.

PfHsp70-1 has intrinsic ATPase activity that influences client refolding activity (16, 31), and both AMK3 and AMK4 inhibit this activity. AMK3 binds within the ATP pocket, like EGCG, to compete with ATP binding (Fig. 4D) and inhibit ATPase activity. On-target N-terminal binding was also confirmed in a cellular context within *P. falciparum*. In contrast, AMK4 competes with peptide binding (Fig. 4C) at the SBD and decreases nucleotide hydrolysis through an allosteric interaction. TSA results with various C-terminal truncations and limited proteolysis experiments further narrowed a proposed AMK4 binding site to the SBDβ sub-domain. It has been previously demonstrated that Hsp70-1 has the ability to bind aliphatic peptide substrates through a conserved interaction within the SBDβ and lid domains (9, 46, 58). Therefore, AMK4 may directly compete with peptide binding or disrupt the SBDβ-lid interaction. An allosteric inhibition of ATPase activity was also observed with the previously reported 116-9e, a molecule that disrupts the J-domain interaction of cytosolic *Pf*Hsp40 co-chaperones (PfYdj1, PfSis1) with *Pf*Hsp70-1, inhibiting the ATPase activity of the complex (59).

Without any optimization, compounds AMK3 and AMK4 exhibited surprising binding selectivity toward *Pf*Hsp70-1 when compared to HSPA1A, with a >25-fold affinity toward *Pf*Hsp70-1. This was unexpected, as heat shock proteins are highly homologous between organisms, particularly within the ATP-binding active site. It has previously been established that the Hsp70-1 substrate binding domain is less conserved, and therefore, selective molecules could be more readily achieved by targeting this region (18, 60, 61). Of note, allosteric modulators exhibit lower affinity to their targets when compared to active-site directed molecules, which is consistent with the higher AMK4 *K*_d_ versus AMK3.

A computational analysis (DeepSite webserver) predicts three possible small-molecule binding sites within the *Pf*Hsp70-1 SBDβ sub-domain, where subsequent docking of AMK4 to these sites suggests modest interactions. Among these, site 1 has the highest predicted affinity and is the known peptide binding site. This largest pocket preferentially binds to aliphatic peptides, which would rationalize the ability of AMK4, but not the N-terminal inhibitors AMK3 or EGCG, to reduce peptide binding. Site 2 is also a compelling possibility for ligand binding. Previously, Leu et al. discovered that a derivative of 2-phenyl-ethynesulfonamide, the aromatic, hydrophobic small molecule PET-16, binds to site 2 within HSPA1A and yields allosteric inhibitory effects (62). PET-16 affects peptide binding, similar to our observations with AMK4 (62). The previously reported HSPA1A inhibitor novolactone is also proposed to bind within site 2 (63). Thus, AMK4 may bind at the same site as the tested peptide substrate (site 1), or the peptide binding pocket may be disrupted through an allosteric interaction (i.e., site 2). It also remains possible that AMK4 has multiple binding sites, including ones that were not detected in our biochemical studies. Future structural studies will be critical for uncovering the ligand-*Pf*Hsp70-1 interaction, as it could confirm the proposed binding site in the SBDβ sub-domain and reveal key molecular interactions that enable selectivity.

It is known that in the ATP-bound state, the NBD and SBD are coupled, yielding weaker affinity for substrate, while in the ADP-bound state, the domains function more independently (7, 60, 64, 65). It is possible that EGCG and AMK3 could mimic the *Pf*Hsp70-1 ADP-bound state, thus uncoupling the NBD and SBD, which would be predicted to have a minimal impact on peptide binding (31, 62). However, future structural and biochemical studies are needed to test these hypotheses.

The molecular basis behind AMK3 species selectivity was investigated with molecular docking and residue interaction analyses. Our data strongly support ligand binding within the ATP-binding pocket, and based on our model, the orientation of the AMK3 terminal imidazole ring appears slightly different between human and *Plasmodium* Hsp70-1. Furthermore, AMK3 bound to *Pf*Hsp70-1 makes two additional predicted hydrogen bonding interactions with the amide carbonyl, adjacent to the indole ring, that are not present in the docked HSPA1A model. These hydrogen bonds, the perpendicular pi-stacking that AMK3 makes with *Pf*Hsp70-1, and the orientation of the indole ring and amide carbonyl could all contribute to a lower *K*d. Moreover, the imidazole ring of AMK3 is likely pH sensitive and could potentially play a role in the observed selectivity. Thus, it will be interesting to complete binding studies at various pH to test this hypothesis. Nevertheless, resolution of the *Pf*Hsp70-1-AMK3 bound structure will be critical to resolve the basis for this species selectivity, where it is likely that multiple factors contribute to the ligand’s higher affinity to the parasite homolog.

In summary, this work identifies small molecules that bind to different *Pf*Hsp70-1 sub-domains with species selectivity. This work deepens our understanding of druggable binding sites within this difficult-to-target protein class. Future structural studies centered on *Pf*Hsp70-1 are critical next steps to resolve these ligand binding sites. Though challenging due to the size and flexibility of *Pf*Hsp70-1, such studies have the potential to shed light on the mechanism of species selectivity and potential allosteric conformational changes induced by inhibitor binding. Our computational-based SAR screen further improved the profile of the NBD-binding molecule AMK3, highlighting compound 12 as a starting point for future optimization. Novel chemical probes that target distinct functional domains of *Pf*Hsp70-1 are needed to interrogate the chaperone’s proposed region-specific functional roles throughout the parasite’s complex lifecycle. Thus, this work lays the groundwork for critical molecular studies, as well as future development of novel antimalarial agents.

## MATERIALS AND METHODS

### General

All reagents were purchased from Sigma-Aldrich (St. Louis, MO) unless otherwise indicated. Prioritized compounds were purchased from AMK1 (Enamine, T5569417), AMK2 (Life Chemicals, F3385-1084), AMK3 (Life Chemicals, F5607-0054), AMK4 (Life Chemicals, F5759-0160). Compounds 1–13 were purchased from Enamine: 1 (Z763964774); 2 (Z1837885405); 3 (Z845681750); 4 (Z764219602); 5 (Z1224938630); 6 (Z810042692); 7 (Z809997974); 8 (PV-002276200143); 9 (Z2095999490); 10 (Z1689559852); 11 (PV-002212004418); 12 (Z2092001983); and 13 (PV-002502670956). All compounds were >90% pure. Homology models were generated using DeepMind’s AlphaFold2 with MMseqs2 (41). Each construct was run separately through the Google Colab notebook, ColabFold: AlphaFold2 w/MMseqs2, with templates and a homooligomeric state of 1 (66). Molecular docking was performed using AutoDock Vina (43, 45). Protein-ligand interactions were visualized using the PLIP webserver, and all visualizations were generated using PyMOL (Schrödinger).

### Expression and purification of protein constructs

*Pf*Hsp70-1 was amplified from cDNA as previously reported (21), and cloned into a pET45b(+) vector in frame with an N-terminal His6-tag. HSPA1A (UniProt access: P0DMV8) (kind gift of Lois Greene, Addgene #15215) was cloned into a pET45b(+) vector in frame with an N-terminal His6-tag. *Pf*Hsp70-1 NBD (1–398) was amplified from full-length *Pf*Hsp70-1 and cloned into a pET45b(+) vector in frame with an N-terminal His6-tag. *Pf*Hsp70-1 SBDβ (398–572), SBDα (548–677), and *Pf*Hsp70-1 C-terminal domain (398–677) were cloned from full-length *Pf*Hsp70-1 into pET21a(+) vectors in frame with an N-terminal T7-tag and C-terminal His6-tag. All constructs are shown in Table S1. All primers were purchased from Eton Bioscience. Plasmids were confirmed by sequencing (Eton Bioscience) before transformation into competent *E. coli* BL21(DE3) cells. For protein expression, cells were grown in four Fernbach flasks containing 1 L of LB media with 100 µg/mL ampicillin at 37°C and 250 rpm. At OD_600_ 0.6, the temperature was reduced to 20°C and protein expression was induced with 0.1 mM IPTG. After 20 h at 20°C, cells were collected by centrifugation (4,300 × *g*, 30 min) and resuspended in lysis buffer (50 mM KH_2_PO_4_, pH 8.0, 200 mM NaCl, 1 mM benzamidine, 5% glycerol (vol/vol), 5 mM β-mercaptoethanol, 10 mM imidazole) supplemented with 1 cOmplete protease inhibitor tablet (Roche). Cells were lysed using an FB120 Sonic Dismembrator (Fisher Scientific) and centrifuged (4,300 × *g*, 3 h). The supernatant was incubated overnight with Ni-NTA resin (Invitrogen) at 4°C. After stringent washes with buffer containing 10 mM imidazole, bound protein was eluted with a linear imidazole gradient (25–150 mM). Fractions containing the protein of interest were exchanged into a low salt buffer (25 mM triethanolamine, pH 8.0, 25 mM NaCl, 1 mM DTT, 5% glycerol [vol/vol]), applied to an anion exchange HQ/10 column (Applied Biosciences), and eluted with a linear salt gradient (25–150 mM). For full-length *Pf*Hsp70-1 and HSPA1A, fractions containing partially purified protein were pooled, re-applied to the anion exchange HQ/10 column (Applied Biosciences), and eluted with a linear salt gradient. Protein purity was assessed by SDS-PAGE, and protein concentration was determined using the Pierce Coomassie Plus Bradford Assay Reagent (ThermoFisher Scientific) and calculated using a BSA standard curve. All proteins were purified to ≥95% homogeneity. Approximate protein yield was 0.5 mg/L for *Pf*Hsp70-1, 1 mg/L for HSPA1A, 2 mg/L for *Pf*Hsp70-1 NBD, 2 mg/L for *Pf*Hsp70-1 SBDβ, 2 mg/L for *Pf*Hsp70-1 SBDα, and 2 mg/L for *Pf*Hsp70-1 C-terminal domain. Protein was concentrated and stored as 25% glycerol stocks at −80°C.

### Thermal shift high-throughput screen

Protein thermal shift assays were performed by first dispensing purified *Pf*Hsp70-1 (3 µM) in assay buffer (10 mM HEPES, pH 8.0, 100 mM KCl, 2 mM MgCl_2_, 0.5 mM DTT) into white 96-well PCR plates (Roche). Compounds (30 µM), the negative DMSO control, or the positive 1 mM ATP control were added to each well, followed by SYPRO orange dye (ThermoFisher Scientific) at 5× concentration. Each well contained 6.7% DMSO in a final volume of 15 µL. Plates were sealed with clear foil, briefly shaken, and centrifuged (1,100 rpm × 2 min). Plates were then analyzed using a LightCycler 480 Instrument II (Roche), from 20°C to 85°C with a 0.06 (°C/s) ramp rate, 10 acquisitions per degree Celsius. Melting temperatures (Tm) were determined by taking the first derivative of each thermal profile (Prism GraphPad). The x ranged from 0.16 to 0.78. Compounds that increased the Tm of *Pf*Hsp70-1 by ≥3.5°C or decreased the Tm of *Pf*Hsp70-1 by ≥−3.5°C were considered hits. A 5-point dose-response curve (0–120 µM) was performed in biological duplicate with all initial screening positive as a secondary screen.

### Hepatocyte cytotoxicity assay

Compounds were tested for cytotoxicity to human cells. Human hepatocyte Huh7 cells were maintained in DMEM supplemented with L-glutamine (Gibco), 10% heat-inactivated fetal bovine serum (vol/vol) (Sigma-Aldrich), and 1% antibiotic-antimycotic (ThermoFisher Scientific) at 37°C with 5% CO_2_. For the assay, Huh7 cells (5,000 cells/well) were seeded into 384-well white microplates (Corning, 3570) and compounds (10 or 30 µM, 1% DMSO) or 1% DMSO were added in triplicate 24 h after seeding. After 48 h, Huh7 viability was assessed using CellTiter-Fluor (Promega) according to the manufacturer’s protocols. Relative fluorescence was measured using an EnVision 2105 plate reader (PerkinElmer). Signal intensity of each well was normalized to the 1% DMSO negative control. Samples were measured across five independent experiments.

### Liver stage *Plasmodium* inhibition assays

Huh7 cells were maintained as described above. Luciferase-expressing *P. berghei* ANKA sporozoites were harvested from freshly dissected salivary glands of *Anopheles* mosquitoes (SporoCore, Athens, Georgia). Huh7 cells (5,000 cells/well) were seeded into 384-well white microplates (Corning, 3570). After 24 h, compounds (0–100 µM) were added in triplicate using a D300e Digital Dispenser (Hewlett-Packard) before infection with *P. berghei* ANKA sporozoites (4,000 sporozoites/well). Each well contained 1% DMSO in a final volume of 30 µL. After 44 h post-infection, Huh7 viability and *Plasmodium* parasite load were assessed using CellTiter-Fluor (Promega) and Bright-Glo (Promega) reagents, respectively, according to the manufacturer’s protocols. Relative fluorescence and luminescence signals were measured using an EnVision 2105 plate reader (PerkinElmer). Signal intensity of each well was normalized to the negative control (1% DMSO) to calculate relative viability. Data were fit to a nonlinear curve (GraphPad Prism) to obtain half-maximal effective concentrations (EC_50_). Samples were measured across three independent experiments.

### Blood stage *Plasmodium* inhibition assays

Asexual blood stage parasite inhibition assays were completed as previously described (67). Briefly, sorbitol (5% wt/vol) synchronized ring-stage *P. falciparum* 3D7 cultures at 2% parasitemia and 1% hematocrit in complete media (10.44 g/L RPMI 1640 [Gibco], 25 mM HEPES, pH 7.2, 0.37 mM hypoxanthine, 24 mM sodium bicarbonate, 0.5% [wt/vol] AlbuMAX II [Gibco], and 25 mg/mL gentamicin) were added to black 96-well non-treated microplates (Corning, 3915). Compounds (0–100 µM) were then dispensed using a D300e Digital Dispenser (Hewlett-Packard). Each well contained 0.5% DMSO in a final volume of 200 µL, and every plate included 0.5% DMSO as a negative control and quinacrine (1 µM, Sigma-Aldrich) as a positive control. After compound addition, assay plates were incubated at 37°C with 3% O_2_, 5% CO_2_, and 92% N_2_ for 72 h. After incubation, 40 μL of lysis buffer (20 mM Tris, pH 7.5, 5 mM EDTA, 0.16% saponin [wt/vol], 1.6% Triton X-100 [vol/vol]) supplemented with SYBR Green I (Invitrogen) at 10× concentration was added to each well. Staining was allowed to proceed for 24 h at room temperature in the dark; afterward, parasite DNA was assessed by measuring SYBR Green I fluorescence using an EnVision 2105 plate reader (PerkinElmer) (Ex: 485; Em: 535). Relative parasite load was calculated by normalizing the fluorescence signal from experimental treatments to the positive and negative controls. EC_50_ values were calculated for each compound (GraphPad Prism), with curves constrained to a bottom limit equal to 0. Samples were measured in duplicate or triplicate per experiment, across 3–5 independent experiments.

### ATPase inhibition assay

ATPase inhibition was performed as described previously with slight modification (68). Briefly, *Pf*Hsp70-1 (0.4 µM) and compound (0–500 µM) in buffer (10 mM HEPES, pH 8.0, 100 mM KCl, 2 mM MgCl_2_, 0.5 mM DTT) were added to a clear 96-well plate and incubated at 37°C for 5 min. ATP (1.5 mM) was then added to a final volume of 80 µL and incubated at 37°C for 30 min. After incubation, 20 µL of working reagent (6.8% sulfuric acid [vol/vol], 61 mM ascorbic acid, 0.2 mM antimonyl tartrate, 4.9 mM ammonium molybdate) was added to each well, and the plate was incubated at room temperature. After 30 min, the absorbance was measured, corresponding to the amount of free phosphate present in solution, at 595 nm using an EnVision 2105 plate reader (PerkinElmer).

### Compound binding by microscale thermophoresis

MST experiments were conducted using a Monolith Pico (NanoTemper Technologies). *Pf*Hsp70-1 and HSPA1A (10 µM) were labeled using NHS-RED second generation (NanoTemper Technologies) following the manufacturer’s protocol. The degree of labeling for each protein was calculated as ~1 using the degree-of-labeling calculator (https://nanotempertech.com/dol-calculator/). Labeled protein (20 nM) was incubated with compound (0–500 µM) in assay buffer (100 mM Na_2_HPO_4_, pH 7.5, 150 mM NaCl, 0.1% Tween-20 [vol/vol], and 5% DMSO [vol/vol]) for 5 min at room temperature before loading into standard Monolith capillaries for MST analysis. Data analysis was performed using the MO. Affinity Analysis software (NanoTemper Technologies) and GraphPad Prism. Each protein was analyzed in at least three independent experiments.

### One-pot SPROX

SPROX was performed as a one-pot analysis similar to previously described (69). Briefly, (+ AMK3) (250 µM), (+ AMK4) (250 µM), and (−) ligand (5% DMSO) samples were distributed into a series of 12 PBS buffers containing increasing urea concentrations between 0.5 and 4.5 M, at equally spaced increments. In total, each of the 12 urea-containing tubes contained 180 µg *E. coli* lysate and was incubated for 2 h at room temperature. After incubation, 1.25 µL of 30% (vol/vol) H_2_O_2_ was added to each urea-containing sample for 3 min before quenching the reactions with 125 µL of 500 mM TCEP. Solutions for each biological replicates were combined. Equal amounts of each ligand sample were combined to give 3 (+ AMK3), 3 (+ AMK4), and 4 (−) ligand samples. Samples were then buffer exchanged into 8 M urea in 0.1 M Tris-HCl, pH 8.5, using a 10 kDa MWCO centrifugal filter, followed by TCEP reduction, MMTS alkylation, trypsin digestion, and TMT 10-Plex labeling. Equal volumes for each labeled sample were combined, and a column clean-up was performed using C18 Macrospin columns (Higgins Analytical Inc.). Methionine-containing peptides were enriched using a Pi^3^ Methionine reagent kit (BioMolecular Technologies Inc.) according to the manufacturer’s protocol. The methionine-enriched sample was dried in a speed vac before LC-MS/MS analysis.

### Blood stage *Plasmodium* parasites isolation for proteomic analysis

Synchronized *P. falciparum* 3D7 parasites were cultured to 10%–15% parasitemia at 1% hematocrit. At the trophozoite stage (22–38 hpi), infected red blood cells were pelleted by centrifugation (400 g) and released from host cells by treatment with 10 volumes of cold 0.03% (wt/vol) saponin (Sigma) in PBS (Gibco). Isolated parasites were subsequently washed four times in 10 volumes of cold PBS at 4,300 × *g* to remove residual host erythrocytes. The parasites were subsequently resuspended in four volumes of PBS supplemented with protease inhibitor cocktail (1 mM AEBSF, 20 μM leupeptin, 10 μM pepstatin A, 500 μM bestatin, and 15 μM E-64) and lysed by sonication. Lysate was clarified by centrifugation at 20,000 × *g,* and protein concentration was determined with Pierce Coomassie Plus Bradford Assay Reagent and a BSA standard curve.

### One-pot STEPP-PP

STEPP-PP was performed as a one-pot analysis as described previously (69). For on-target binding studies, (+ AMK3) (250 µM), and (−) ligand (5% DMSO) samples were added to 12 urea-containing buffers (0.5–4.95 M) in 0.37 M increments. Each sample contained 100 µg *P. falciparum* lysate. For *E. coli Pf*Hsp70-1 overexpression studies, (+ AMK3) (250 µM), (+ AMK4) (250 µM), and (−) ligand (5% DMSO) samples were added to 12 urea-containing buffers (0.5–4.5 M) in 0.33 M increments. Each sample contained 100 µg *E. coli* lysate. Samples were incubated for 2 h at room temperature. After incubation, 2 µg thermolysin was added to each sample and allowed to react for 1 min at room temperature, followed by quenching with 60 µL of solution 1 (0.2 M EDTA, 8 M urea, pH 8.0). Equal volumes of the (+ AMK3) and (−) ligand were combined to give 3 (+ AMK3) samples and 3 (−) ligand samples (for *P. falciparum* lysate experiments). For *E. coli* lysate experiments, equal volumes of the (+ AMK3), (+ AMK4), and (−) ligand were combined to give 3 (+ AMK3) samples, 3 (+ AMK4) samples, and 4 (−) ligand samples. For all labeling, each sample was reacted with 1.5 mM TCEP for 1 h at 30°C, followed by 3 mM MMTS for 15 min at room temperature. Samples were then labeled with a TMT kit according to the manufacturer’s protocol. The TMT-labeled samples were combined, lyophilized, dissolved in 2% (vol/vol) TFA, and desalted on C18 Macrospin columns (Higgins Analytical Inc.). Samples were then lyophilized, dissolved in 0.1 M TEAB (pH 8.5), and digested with trypsin protease (1:50) overnight at 37°C. Samples were added to NHS-activated agarose resin (150:1) along with 50 μL of 0.5 M NaCl. Samples were allowed to react for 1.5 h at room temperature, acidified with 2% (vol/vol) TFA, and then transferred to C18 Macrospin columns prior to LC-MS/MS.

### LC-MS/MS and proteomic data analysis

LC-MS/MS spectra were acquired using an Easy-nLC 1200 System (ThermoFisher Scientific) coupled to a Thermo Orbitrap Exploris 480 mass spectrometer (ThermoFisher Scientific). Dried peptides were resuspended to 1 µg/µL with 0.1% TFA, 2% ACN in H_2_O. One microliter of the sample was injected into the Easy-nLC 1200 System. Separation was performed using an Easy-Spray HPLC 75 µm × 150 mm reversed C18 column (ThermoFisher Scientific) with a 2 µm particle size. Peptide elution was performed using a linear gradient (4%–35% ACN with 0.1% formic acid at 400 nL/min). MS data were collected using MS1 at 120K and MS2 at 45K resolution. For MS1, the AGC target was 3.0 × 10^6^ ions with a max injection time set to auto mode. For MS2, the AGC target was 3.0 × 10^5^ ions with a max injection time of 105 ms. The HCD collision energy was 36%, and the scan range was from 200 to 2,000 *m*/*z*. The dynamic exclusion duration was 45 s and the isolation window was 1.2 *m*/*z*. Proteome Discoverer 2.3 (ThermoFisher Scientific) was used to search the raw LC-MS/MS files against the UniProt Knowledge base. The enzyme set was trypsin (full), allowing for up to two missed cleavages for SPROX and trypsin (semi) with at maximum three missed cleavages allowed for STEPP-PP. A normalization factor for each (+ AMK3), (+AMK4), and (−) ligand sample was calculated for each biological replicate using the reporter ion intensities from the wild-type methionine-containing peptides (SPROX) and semitryptic peptides (STEPP-PP). For each identified peptide, a ratio was calculated of the observed reporter ion intensities in the (+) ligands to the (−) ligand sample for each biological replicate. This ratio was divided by the normalization factor, which was then log2 transformed, averaged, and tested for significance. Experiments were completed with at least three biological replicates.

### Circular dichroism spectroscopy

CD spectra were collected with a JASCO J-1700 circular dichroism spectrometer. Spectra were collected over a wavelength range of 350 to 190 nm at 25°C in 10 mM sodium phosphate buffer, pH 7, 50 mM NaCl. Protein concentrations were 1–5 µM and all spectra were subtracted from a buffer blank. CD millidegree signal was subsequently converted to molar ellipticity.

### Thermal shift assay on *Pf*Hsp70-1 truncations

Thermal shift assays were performed on *Pf*Hsp70-1 truncations by dispensing purified protein (3 µM) in assay buffer (10 mM HEPES, pH 8.0, 100 mM KCl, 2 mM MgCl_2_, 0.5 mM DTT) into white 96-well plates (Roche). Compounds (0–200 µM) or DMSO were added to each well, followed by SYPRO Orange dye (ThermoFisher Scientific) at a concentration of 5×. Each well contained 6.7% DMSO in a final volume of 15 µL. Plates were sealed with clear foil, briefly shaken, and centrifuged (1,100 rpm × 2 min) before analysis using a LightCycler 480 Instrument II (Roche). The temperature changed from 20°C to 85°C with a 0.06 (°C/s) ramp rate, 10 acquisitions per degree Celsius. Tm’s were determined by taking the first derivative of each thermal profile (GraphPad Prism). Experiments were completed in technical duplicate with at least three biological replicates.

### Limited proteolysis assay

Limited proteolysis experiments were performed using 5 µg purified *Pf*Hsp70-1 SBDβ or SBDα. Proteins were first incubated with AMK4 (200 µM) or DMSO (5%) for 30 min at 4°C before trypsin (0.3 µL) (Sigma-Aldrich) at 1 mg/mL in 50 mM acetic acid was added. Aliquots were taken at different time points post-trypsin addition and immediately boiled in SDS-PAGE loading dye. Samples were resolved by SDS-PAGE and visualized with Coomassie staining. Band intensity analysis was completed with ImageJ to determine the percent intact protein remaining at each time point, where bands were normalized to the sample without trypsin addition. Two independent biological replicates were performed for *Pf*Hsp70-1 SBDβ and SBDα.

### Fluorescence polarization assay to evaluate peptide binding

FITC-NR (NRLLLTG) (46) peptide was synthesized (GenScript) and diluted to 2 mM in assay buffer (10 mM HEPES, pH 8.0, 100 mM KCl, 2 mM MgCl_2_, 0.5 mM DTT). Peptide (10 nM) was then added to full-length *Pf*Hsp70-1 (0–40 µM) and truncations (NBD, SBDα, SBDβ) in a black 384-well microplate (Corning, 3575) to a final volume of 40 µL (46). Each plate included wells without protein as an unbound tracer control. Assay plates were then incubated overnight in the dark at 4°C with rocking to promote binding. Plates were brought to room temperature before fluorescence polarization was measured using an Envision 2105 plate reader (PerkinElmer). The excitation was at 480 nm and emission was recorded at 535 nm. The normalized fluorescence polarization of FITC-peptide bound to *Pf*Hsp70-1 was calculated by normalizing data to the unbound tracer control. Data were fit to a nonlinear curve (GraphPad Prism) to determine the *K*_d(app)_ of 1.3 µM. After establishing peptide affinity to the protein, compounds were investigated for their potential impact on peptide binding. *Pf*Hsp70-1 (1.3 µM) was incubated with EGCG, AMK3, and AMK4 at 10 or 100 µM, or 2.5% DMSO for 30 min at 4°C. Peptide (10 nM) was then added to each well, and plates were incubated overnight at 4°C with rocking to promote binding equilibrium. Fluorescence polarization was then measured as described above. The percent FITC-peptide bound to *Pf*Hsp70-1 was calculated by normalizing data to unbound tracer controls. Experiments were completed with at least three biological replicates in technical duplicates.

### Fluorescence polarization ATP competitive binding titration assay

Fluorescein-12-ATP (F12-ATP) was purchased (PerkinElmer) and diluted in assay buffer (20 mM Tris, pH 6.5, 100 mM NaCl, 0.01% Tween-20). To measure the binding affinity of *Pf*Hsp70-1 NBD to F12-ATP, increasing concentrations of purified *Pf*Hsp70-1 NBD (0.0316–3160 nM) were added to assay buffer in a black 384-well microplate (Corning, 3575) to a final volume of 24 µL, containing 4% DMSO. F12-ATP was then added (5 nM) to each well, for a final well volume of 25 µL, and plates were incubated at room temperature in the dark for 10 min. Fluorescence polarization was measured using an Envision 2105 plate reader (PerkinElmer) (48). The excitation was 480 nm, and emission was recorded at 535 nm. Data were fit to a nonlinear curve (GraphPad Prism) to determine the *K*_d(app)_ value. For F12-ATP displacement assays, 100 nM *Pf*Hsp70-1 NBD (corresponding to the first point at 100% binding) was incubated with increasing concentrations of EGCG, AMK3, and AMK4 (0.00316–100 µM) at a final DMSO concentration of 4% in assay buffer in a black 384-well microplate (Corning, 3575) to a final volume of 24 µL. F12-ATP was then added (5 nM) to each well, for a final well volume of 25 µL, and plates were incubated at room temperature in the dark for 10 min. Fluorescence polarization was then measured as described above (48). The percent F12-ATP bound to *Pf*Hsp70-1 NBD was calculated by normalizing data to unbound and fully bound tracer controls. Wells containing no *Pf*Hsp70-1 NBD or F12-ATP and increasing compound concentrations were used to correct for compound auto fluorescence. Experiments were completed with at least three biological replicates in technical triplicate.

### AutoDock Vina docking

A *Pf*Hsp70-1 homology model generated using Alphafold2 and the crystal structure of HSPA1A NBD bound to ADP (PDB ID: 3ATV) was used for ligand docking studies (41). AutoDockTools 1.5.6 was used to resolve hydrogen atoms and convert to PDBQT (43). All ligands were converted to PDBQT using PyRx. For *Pf*Hsp70-1 and HSPA1A NBD, the grid box was placed within the active site, and the x, y, and z grid points were set at 25 while the grid point spacing was 0.375 Å. For *Pf*Hsp70-1 SBDβ, the grid box was placed at each predicted binding site individually. AutoDock Vina was used to dock all ligands using an exhaustiveness of 32 (43, 45). Receptor-ligand docked structures were visualized in PyMOL and PLIP (44).

### Molecular dynamic simulations and SAR selection process

*Pf*Hsp70-1 NBD homology model was generated using Alphafold2 (41, 66). MD simulations with ligand bound to the *Pf*Hsp70-1 NBD were then performed with AMBER20 (70–73). Enamine similarity search was used to generate a list of 200 structurally similar compounds. Top 25 docked structures of AMK3 SAR ligands were subjected to duplicate 100 ns MD simulations. Ligands were first converted to PDB using PyMOL. The Reduce command was used to add all hydrogens to *Pf*Hsp70-1 NBD, followed by pdb4amber to clean and prepare the structure. Antechamber was used to parameterize all ligand files using the general Amber force field (74, 75). Tleap was used to create the ligand-receptor complex. The ff14SB force field was applied to the complex, which was immersed into a periodic TIP3P water box neutralized with NaCl, extended 8 Å from any solute atom (76). A three-step minimization was performed for each protein, and the Particle Mesh Ewald method was applied to handle long-range electrostatics (77). First, 5,000 cycles of steepest descent and 5,000 cycles of conjugate gradient with 50 kcal/mol/Å² restraint on ligand and backbone carbons, totaling 10,000 cycles of minimization, were performed. Next, the same 10,000 minimization cycles were performed with a 10 kcal/mol/Å² restraint on backbone carbons. Then 10,000 cycles of minimization were performed without protein, including 5,000 cycles of steepest descent and 5,000 cycles of conjugate gradient. The temperature of each system was elevated from 0 to 300 K in 50 ps with a 2.0 kcal/mol/Å² restraint on backbone carbons. Followed by 50 ps of density equilibration without backbone restraint. Finally, a 100 ns NPT (T = 300 K and P = 1 atm) MD simulation was performed with a 2 fs time step. The SHAKE algorithm was used to handle all hydrogen-bonded atoms (78). Each simulation was repeated with a different random seed to support observations. Ligand RMSD was calculated using CPPTRAJ (79). For our SAR study, we performed a structural similarity search on the Enamine REAL compound database; from this, we selected the top 200 compounds. After docking, the top 25 docked structures were subjected to MD simulations. For ligand-complex validation, the last frame of each simulation was visually inspected to evaluate the stability, orientation, and structure of the protein-ligand complexes. Similar to other studies, we selected complexes that had limited ligand movement (<4 Å average) with the ligand remaining inside the active site throughout the simulations. From this, 15 of the top 25 SAR ligands passed our stringent analysis, while 10 of the 25 failed. Of the 15 passing compounds, 13 were readily available for purchase and were received.

## SUPPLEMENTAL MATERIAL

Supplemental material aac.00466-26-s0001.pdf Table S1; Fig. S1 to S15.

ASM does not own the copyrights to Supplemental Material that may be linked to, or accessed through, an article. The authors have granted ASM a non-exclusive, world-wide license to publish the Supplemental Material files. Please contact the corresponding author directly for reuse.
