# Supplementary material for: Development of domain-specific probes of Plasmodium falciparum heat shock protein 70-1
Source: Antimicrob Agents Chemother. 2026 Jul 2;70(8):e00466-26. doi: 10.1128/aac.00466-26 (PMC13435959; doi:10.1128/aac.00466-26)
Supplement: Supplementary file 1 [file aac.00466-26-s0001.pdf]

Supplemental Material

Development of Domain-Specific Probes of *Plasmodium falciparum* Heat Shock Protein 70-1

Aaron M. Keeler,[1] Gaini Ibrasheva,[1] Elizabeth G. Boger,[1] Yueqi Chen,[1] Porter E. Petruzziello,[1] Erin A. Schroeder,[2] Christopher R. Mansfield,[2] Kayla Sylvester,[2] Cameron D. Fleming,[1] Philip F. Hughes,[3] Timothy A. J. Haystead,[3] Michael C. Fitzgerald,[1] Emily R. Derbyshire[1,2*]

[1]Department of Chemistry, Duke University, Durham, NC 27708, USA

[2]Department of Molecular Genetics and Microbiology, Duke University Medical Center, Durham, NC 27710, USA

[3]Department of Pharmacology and Cancer Biology, Duke University Medical Center, Durham, NC 27710, USA

*Correspondence: emily.derbyshire@duke.edu

Supplemental Table

Table S1. Amino acid and nucleotide sequences for all plasmid constructs.

Name	Sequence
pET45b 6xHis-*Pf*Hsp70-1	MAHHHHHHVGTGSMASAKGSKPNLPESNIAIGIDLGTTYSCVGVWRNENVDIIANDQGNRTTPSY<br>VAFTDTERLIGDAAKNQVARNPENTVFDAKRLIGRKFTESSVQSDMKHWPFTVKSGVDEKPMIEV<br>TYQGEKKLFHPEEISSMVLQKMKENAEAFLGKSIKNAVITVPAYFNDSQRQATKDAGTIAGLNVMR<br>IINEPTAAAIAYGLHKKGKGEKNILIFDLGGGTFDVSLLTIEDGIFEVKATAGDTHLGGEDFDNRLVN<br>FCVEDFKRKNRGKDLSKNSRALRRLRTQCERAKRTLSSSTQATIEIDSLFEGIDYSVTVSRARFEEL<br>CIDYFRDTLIPVEKVLKDAMMDKKSVHEVVLVGGSTRIPKIQTLIKEFFNGKEACRSINPDEAVAYG<br>AAVQAAILSGDQSNAVQDLLLLDVCSLSLGLETAGGVMTKLIERNTTIPAKKSQIFTTYADNQPGVLI<br>QVYEGERALTKDNNLLGKFHLDGIPPAPRKVPQIEVTFDIDANGILNVTAVEKSTGKQNHITITND<br>KGRLSQDEIDRMVNDAEKYKAEDEENRKRIEARNSLENYCYGVKSSLEDQKIKEKLQPAEIETCM<br>KTITTILEWLEKNQLAGKDEYEAKQKEAESVCAPIMSKIYQDAAGAAGGMPGGMPGGMPGGMPG<br>GMNFPGGMPGAGMPGNAPAGSGPTVEEVDT*
	ATGGCACATCACCACCACCATCACGTGGGTACCGGTTCGATGGCTAGTGCAAAAGGTTCAAAACCA<br>AATTTACCAGAATCCAATATCGCTATTGGAATTGATTTAGGTACTACTTATTCTTGTGTTGGTGTAT<br>GGAGAAATGAAAATGTAGATATTATTGCTAATGACCAAGGTAATAGAACAACCCCATCTTATGTTG<br>CTTTCACCGATACCGAAAGATTAATTGGAGATGCTGCTAAAAACCAAGTAGCTAGGAATCCAGAAA<br>ATACAGTATTTGATGCTAAGAGATTAATTGGAAGAAAATTTACAGAATCATCAGTACAAAGTGATA<br>TGAAACATTGGCCATTCACTGTTAAATCAGGTGTTGATGAGAAACCAATGATTGAAGTTACCTATC<br>AAGGAGAAAAGAAATTATTCCATCCAGAAGAAATTTCTTCTATGGTATTACAAAAAATGAAAGAA<br>AATGCTGAAGCATTTTTAGGAAAATCTATAAAGAATGCTGTCATTACCGTTCCAGCTTATTTTAAC<br>GATTCACAAAGACAAGCTACTAAAGATGCTGGTACAATTGCAGGATTAAATGTTATGAGAATTATT<br>AATGAACCTACTGCAGCTGCTATTGCATATGGTTTACACAAAAAAGGAAAAGGTGAAAAGAACAT<br>TTTAATTTTCGACTTAGGAGGAGGTACATTTGATGTATCATTATTAACTATTGAAGATGGTATTTT<br>TGAAGTAAAAGCTACTGCTGGTGATACTCATTTAGGTGGTGAAGATTTCGATAACAGATTAGTAAA<br>TTTCTGTGTTGAAGATTTCAAAAGAAAAAACAGAGGTAAAGATTTATCAAAAAATAGTAGAGCCT<br>TAAGAAGATTAAGAACACAATGTGAAAGAGCAAAACGTACTTTATCATCATCTACACAAGCTACAA<br>TTGAAATAGATTCCTTATTTGAAGGTATTGATTACAGTGTTACTGTAAGTAGAGCAAGATTTGAAG<br>AATTATGTATCGACTATTTCCGTGATACTTTAATTCCAGTAGAAAAAGTTTTAAAGGATGCTATGA<br>TGGATAAAAAAAGTGTACATGAAGTTGTTTTAGTTGGTGGTTCTACAAGAATTCCAAAAATCCAA<br>ACTTTAATAAAAGAATTCTTTAATGGTAAAGAAGCATGCAGATCAATTAACCCAGATGAAGCTGT<br>TGCATATGGTGCAGCTGTACAAGCAGCCATTTTATCTGGTGACCAATCAAATGCTGTCCAAGATTT<br>ATTATTATTAGATGTTTGCTCCTTATCATTAGGCTTAGAAACTGCTGGTGGTGTTATGACCAAATT<br>AATTGAAAGAAACACAACCATACCTGCTAAAAAGAGTCAAATCTTTACTACTTATGCTGATAACCA<br>ACCAGGTGTCTTAATTCAAGTATATGAAGGTGAAAGAGCCTTAACCAAAGATAACAATTTATTAGG<br>AAAATTTCACTTAGATGGTATTCCACCTGCACCAAGAAAGGTACCACAAATCGAAGTTACATTCGA<br>TATCGATGCTAACGGTATCTTAAACGTTACGGCTGTAGAAAAATCCACTGGTAAACAAAACCATAT<br>TACAATTACCAACGACAAAGGAAGATTATCTCAAGATGAAATTGATCGTATGGTTAATGATGCTGA<br>AAAATACAAAGCAGAAGATGAAGAAAACAGAAAAAGAATCGAAGCAAGAAACAGCCTTGAAAAT<br>TACTGCTATGGAGTTAAAAGCTCATTAGAAGACCAAAAAATTAAAGAAAAATTACAACCAGCTGA<br>AATTGAAACATGTATGAAAACTATTACAACCATACTTGAATGGTTAGAAAAAAACCAACTTGCTG<br>GAAAAGATGAATATGAAGCCAAACAAAAAGAAGCAGAATCGGTTTGTGCTCCAATTATGTCTAAA<br>ATCTATCAAGATGCTGCTGGTGCAGCCGGTGGTATGCCAGGAGGTATGCCCGGTGGAATGCCAGGT<br>GGAATGCCAGGTGGTATGAATTTCCCAGGAGGTATGCCCGGAGCAGGAATGCCAGGAAATGCCCC<br>AGCTGGAAGTGGACCAACAGTTGAAGAAGTTGATACCTAG
pET45b 6xHis-*Pf*Hsp70-1 NBD	MAHHHHHHVGTGSNDDDDKSPDPMASAKGSKPNLPESNIAIGIDLGTTYSCVGVWRNENVDIIA<br>NDQGNRTTPSYVAFTDTERLIGDAAKNQVARNPENTVFDAKRLIGRKFTESSVQSDMKHWPFTV<br>KSGVDEKPMIEVTYQGEKKLFHPEEISSMVLQKMKENAEAFLGKSIKNAVITVPAYFNDSQRQATK<br>DAGTIAGLNVMRIINEPTAAAIAYGLHKKGKGEKNILIFDLGGGTFDVSLLTIEDGIFEVKATAGDTH<br>LGGEDFDNRLVNFCVEDFKRKNRGKDLSKNSRALRRLRTQCERAKRTLSSSTQATIEIDSLFEGIDY<br>SVTVSRARFEELCIDYFRDTLIPVEKVLKDAMMDKKSVHEVVLVGGSTRIPKIQTLIKEFFNGKEAC<br>RSINPDEAVAYGAAVQAAILSGDQS*

	ATGGCACATCACCACCACCATCACGTGGGTACCGGTTCGAATGATGACGACGACAAGAGTCCGGAT<br>CCCATGGCTAGTGCAAAAGGTTCAAAACCAAATTTACCAGAATCCAATATCGCTATTGGAATTGAT<br>TTAGGTACTACTTATTCTTGTGTTGGTGTATGGAGAAATGAAAATGTAGATATTATTGCTAATGAC<br>CAAGGTAATAGAACAACCCCATCTTATGTTGCTTTCACCGATACCGAAAGATTAATTGGAGATGCT<br>GCTAAAAACCAAGTAGCTAGGAATCCAGAAAATACAGTATTTGATGCTAAGAGATTAATTGGAAG<br>AAAATTTACAGAATCATCAGTACAAAGTGATATGAAACATTGGCCATTCACTGTTAAATCAGGTGT<br>TGATGAGAAACCAATGATTGAAGTTACCTATCAAGGAGAAAAGAAATTATTCCATCCAGAAGAAA<br>TTTCTTCTATGGTATTACAAAAAATGAAAGAAAATGCTGAAGCATTTTTAGGAAAATCTATAAAG<br>AATGCTGTCATTACCGTTCCAGCTTATTTTAACGATTCACAAAGACAAGCTACTAAAGATGCTGGT<br>ACAATTGCAGGATTAAATGTTATGAGAATTATTAATGAACCTACTGCAGCTGCTATTGCATATGGT<br>TTACACAAAAAAGGAAAAGGTGAAAAGAACATTTTAATTTTCGACTTAGGAGGAGGTACATTTGA<br>TGTATCATTATTAACTATTGAAGATGGTATTTTTGAAGTAAAAGCTACTGCTGGTGATACTCATTT<br>AGGTGGTGAAGATTTCGATAACAGATTAGTAAATTTCTGTGTTGAAGATTTCAAAAGAAAAAACA<br>GAGGTAAAGATTTATCAAAAAATAGTAGAGCCTTAAGAAGATTAAGAACACAATGTGAAAGAGCA<br>AAACGTACTTTATCATCATCTACACAAGCTACAATTGAAATAGATTCCTTATTTGAAGGTATTGAT<br>TACAGTGTTACTGTAAGTAGAGCAAGATTTGAAGAATTATGTATCGACTATTTCCGTGATACTTTA<br>ATTCCAGTAGAAAAAGTTTTAAAGGATGCTATGATGGATAAAAAAAGTGTACATGAAGTTGTTTT<br>AGTTGGTGGTTCTACAAGAATTCCAAAAATCCAAACTTTAATAAAAGAATTCTTTAATGGTAAAG<br>AAGCATGCAGATCAATTAACCCAGATGAAGCTGTTGCATATGGTGCAGCTGTACAAGCAGCCATTT<br>TATCTGGTGACCAATCATGA
pET45b<br>6xHis-<br>HSPA1A	MAHHHHHHVGTGSMAKAAAIGIDLGTTYSCVGVFQHGKVEIIANDQGNRTTPSYVAFTDTERLIG<br>DAAKNQVALNPQNTVFDAKRLIGRKFGDPVVQSDMKHWPFQVINDGDKPKVQVSYKGETKAFYP<br>EEISSMVLTKMKEIAEAYLGYPVTNAVITVPAYFNDSQRQATKDAGVIAGLNVLRIINEPTAAAIAYG<br>LDRTGKGERNVLIFDLGGGTFDVSILTIDDGIFEVKATAGDTHLGGEDFDNRLVNHFVEEFKRKHK<br>KDISQNKRAVRRLRTACERAKRTLSSSTQASLEIDSLFEGIDFYTSITRARFEELCSDLFRSTLEPVE<br>KALRDAKLDKAQIHDLVLVGGSTRIPKVQKLLQDFFNGRDLNKSINPDEAVAYGAAVQAAILMGDK<br>SENVQDLLLLDVAPLSLGLETAGGVMTALIKRNSTIPTKQTQIFTTYSDNQPGVLIQVYEGERAMTK<br>DNNLLGRFELSGIPPAPRGVPQIEVTFDIDANGILNVTATDKSTGKANKITITNDKGRLSKEEIERM<br>VQEAEKYKAEDEVQRERVSAKNALESYAFNMKSAVEDEGLKGKISEADKKKVLDKCQEVISWLDA<br>NTLAEKDEFEHKRKELEQVCNPIISGLYQGAGGPGPGGFGAQGPKGGSGSGPTIEEVD*
	ATGGCACATCACCACCACCATCACGTGGGTACCGGTTCGATGGCCAAAGCCGCGGCGATCGGCATC<br>GACCTGGGCACCACCTACTCCTGCGTGGGGGTGTTCCAACACGGCAAGGTGGAGATCATCGCCAAC<br>GACCAGGGCAACCGCACCACCCCCAGCTACGTGGCCTTCACGGACACCGAGCGGCTCATCGGGGAT<br>GCGGCCAAGAACCAGGTGGCGCTGAACCCGCAGAACACCGTGTTTGACGCGAAGCGGCTGATCGG<br>CCGCAAGTTCGGCGACCCGGTGGTGCAGTCGGACATGAAGCACTGGCCTTTCCAGGTGATCAACG<br>ACGGAGACAAGCCCAAGGTGCAGGTGAGCTACAAGGGGGAGACCAAGGCATTCTACCCCGAGGAG<br>ATCTCGTCCATGGTGCTGACCAAGATGAAGGAGATCGCCGAGGCGTACCTGGGCTACCCGGTGACC<br>AACGCGGTGATCACCGTGCCGGCCTACTTCAACGACTCGCAGCGCCAGGCCACCAAGGATGCGGGT<br>GTGATCGCGGGGCTCAACGTGCTGCGGATCATCAACGAGCCCACGGCCGCCGCCATCGCCTACGGC<br>CTGGACAGAACGGGCAAGGGGGAGCGCAACGTGCTCATCTTTGACCTGGGCGGGGGCACCTTCGA<br>CGTGTCCATCCTGACGATCGACGACGGCATCTTCGAGGTGAAGGCCACGGCCGGGGACACCCACCT<br>GGGTGGGGAGGACTTTGACAACAGGCTGGTGAACCACTTCGTGGAGGAGTTCAAGAGAAAACAC<br>AAGAAGGACATCAGCCAGAACAAGCGAGCCGTGAGGCGGCTGCGCACCGCCTGCGAGAGGGCCAA<br>GAGGACCCTGTCGTCCAGCACCCAGGCCAGCCTGGAGATCGACTCCCTGTTTGAGGGCATCGACTT<br>CTACACGTCCATCACCAGGGCGAGGTTCGAGGAGCTGTGCTCCGACCTGTTCCGAAGCACCCTGGA<br>GCCCGTGGAGAAGGCTCTGCGCGACGCCAAGCTGGACAAGGCCCAGATTCACGACCTGGTCCTGG<br>TCGGGGGCTCCACCCGCATCCCCAAGGTGCAGAAGCTGCTGCAGGACTTCTTCAACGGGCGCGACC<br>TGAACAAGAGCATCAACCCCGACGAGGCTGTGGCCTACGGGGCGGCGGTGCAGGCGGCCATCCTG<br>ATGGGGGACAAGTCCGAGAACGTGCAGGACCTGCTGCTGCTGGACGTGGCTCCCCTGTCGCTGGG<br>GCTGGAGACGGCCGGAGGCGTGATGACTGCCCTGATCAAGCGCAACTCCACCATCCCCACCAAGCA<br>GACGCAGATCTTCACCACCTACTCCGACAACCAACCCGGGGTGCTGATCCAGGTGTACGAGGGCGA<br>GAGGGCCATGACGAAAGACAACAATCTGTTGGGGCGCTTCGAGCTGAGCGGCATCCCTCCGGCCC<br>CCAGGGGCGTGCCCCAGATCGAGGTGACCTTCGACATCGATGCCAACGGCATCCTGAACGTCACGG<br>CCACGGACAAGAGCACCGGCAAGGCCAACAAGATCACCATCACCAACGACAAGGGCCGCCTGAGC<br>AAGGAGGAGATCGAGCGCATGGTGCAGGAGGCGGAGAAGTACAAAGCGGAGGACGAGGTGCAGCG

	CGAGAGGGTGTCAGCCAAGAACGCCCTGGAGTCCTACGCCTTCAACATGAAGAGCGCCGTGGAGG<br>ATGAGGGGCTCAAGGGCAAGATCAGCGAGGCCGACAAGAAGAAGGTGCTGGACAAGTGTCAAGA<br>GGTCATCTCGTGGCTGGACGCCAACACCTTGGCCGAGAAGGACGAGTTTGAGCACAAGAGGAAGG<br>AGCTGGAGCAGGTGTGTAACCCCATCATCAGCGGACTGTACCAGGGTGCCGGTGGTCCCGGGCCTG<br>GGGGCTTCGGGGCTCAGGGTCCCAAGGGAGGGTCTGGGTCAGGCCCCACCATTGAGGAGGTAGAT<br>TAG
pET21a(+) T7-Tag-*Pf*Hsp70-1 SBDα + DR-6xHis	MASMTGGQQMGRGSRIEARNSLENYCYGVKSSLEDQKIKEKLQPAEIETCMKTITTILEWLEKNQ<br>LAGKDEYEAKQKEAESVCAPIMSKIYQDAAGAAGGMPGGMPGGMPGGMPGGMNFPGGMPGAG<br>MPGNAPAGSGPTVEEVDKLAAALEHHHHHH*
	ATGGCTAGCATGACTGGTGGACAGCAAATGGGTCGCGGATCCAGAATCGAAGCAAGAAACAGCCT<br>TGAAAATTACTGCTATGGAGTTAAAAGCTCATTAGAAGACCAAAAAATTAAAGAAAAATTACAAC<br>CAGCTGAAATTGAAACATGTATGAAAACTATTACAACCATACTTGAATGGTTAGAAAAAAACCAA<br>CTTGCTGGAAAAGATGAATATGAAGCCAAACAAAAAGAAGCAGAATCGGTTTGTGCTCCAATTAT<br>GTCTAAAATCTATCAAGATGCTGCTGGTGCAGCCGGTGGTATGCCAGGAGGTATGCCCGGTGGAAT<br>GCCAGGTGGAATGCCAGGTGGTATGAATTTCCCAGGAGGTATGCCCGGAGCAGGAATGCCAGGAA<br>ATGCCCCAGCTGGAAGTGGACCAACAGTTGAAGAAGTTGATAAGCTTGCGGCCGCACTCGAGCAC<br>CACCACCACCACCACTGA
pET21a(+) T7-Tag-*Pf*Hsp70-1 SBDβ-6xHis	MASMTGGQQMGRGSSNAVQDLLLLDVCSLSLGLETAGGVMTKLIERNTTIPAKKSQIFTTYADNQP<br>GVLIQVYEGERALTKDNNLLGKFHLDGIPPAPRKVPQIEVTFDIDANGILNVTAVEKSTGKQNHITI<br>TNDKGRLSQDEIDRMVNDAEKYKAEDEENRKRIEARNSLENYCYGVKSSLEDQKIKKLAAALEHH<br>HHHH*
	ATGGCTAGCATGACTGGTGGACAGCAAATGGGTCGCGGATCCTCAAATGCTGTCCAAGATTTATTA<br>TTATTAGATGTTTGCTCCTTATCATTAGGCTTAGAAACTGCTGGTGGTGTTATGACCAAATTAATT<br>GAAAGAAACACAACCATACCTGCTAAAAAGAGTCAAATCTTTACTACTTATGCTGATAACCAACCA<br>GGTGTCTTAATTCAAGTATATGAAGGTGAAAGAGCCTTAACCAAAGATAACAATTTATTAGGAAA<br>ATTTCACTTAGATGGTATTCCACCTGCACCAAGAAAGGTACCACAAATCGAAGTTACATTCGATAT<br>CGATGCTAACGGTATCTTAAACGTTACGGCTGTAGAAAAATCCACTGGTAAACAAAACCATATTAC<br>AATTACCAACGACAAAGGAAGATTATCTCAAGATGAAATTGATCGTATGGTTAATGATGCTGAAA<br>AATACAAAGCAGAAGATGAAGAAAACAGAAAAAGAATCGAAGCAAGAAACAGCCTTGAAAATTA<br>CTGCTATGGAGTTAAAAGCTCATTAGAAGACCAAAAAATTAAAAAGCTTGCGGCCGCACTCGAGC<br>ACCACCACCACCACCACTGA
pET21a(+) T7-Tag-*Pf*Hsp70-1 C-terminal Domain-6xHis	MASMTGGQQMGRGSSNAVQDLLLLDVCSLSLGLETAGGVMTKLIERNTTIPAKKSQIFTTYADNQP<br>GVLIQVYEGERALTKDNNLLGKFHLDGIPPAPRKVPQIEVTFDIDANGILNVTAVEKSTGKQNHITI<br>TNDKGRLSQDEIDRMVNDAEKYKAEDEENRKRIEARNSLENYCYGVKSSLEDQKIKEKLQPAEIET<br>CMKTITTILEWLEKNQLAGKDEYEAKQKEAESVCAPIMSKIYQDAAGAAGGMPGGMPGGMPGGM<br>PGGMNFPGGMPGAGMPGNAPAGSGPTVEEVDKLAAALEHHHHHH*
	ATGGCTAGCATGACTGGTGGACAGCAAATGGGTCGCGGATCCTCAAATGCTGTCCAAGATTTATTA<br>TTATTAGATGTTTGCTCCTTATCATTAGGCTTAGAAACTGCTGGTGGTGTTATGACCAAATTAATT<br>GAAAGAAACACAACCATACCTGCTAAAAAGAGTCAAATCTTTACTACTTATGCTGATAACCAACCA<br>GGTGTCTTAATTCAAGTATATGAAGGTGAAAGAGCCTTAACCAAAGATAACAATTTATTAGGAAA<br>ATTTCACTTAGATGGTATTCCACCTGCACCAAGAAAGGTACCACAAATCGAAGTTACATTCGATAT<br>CGATGCTAACGGTATCTTAAACGTTACGGCTGTAGAAAAATCCACTGGTAAACAAAACCATATTAC<br>AATTACCAACGACAAAGGAAGATTATCTCAAGATGAAATTGATCGTATGGTTAATGATGCTGAAA<br>AATACAAAGCAGAAGATGAAGAAAACAGAAAAAGAATCGAAGCAAGAAACAGCCTTGAAAATTA<br>CTGCTATGGAGTTAAAAGCTCATTAGAAGACCAAAAAATTAAAGAAAAATTACAACCAGCTGAAA<br>TTGAAACATGTATGAAAACTATTACAACCATACTTGAATGGTTAGAAAAAAACCAACTTGCTGGA<br>AAAGATGAATATGAAGCCAAACAAAAAGAAGCAGAATCGGTTTGTGCTCCAATTATGTCTAAAAT<br>CTATCAAGATGCTGCTGGTGCAGCCGGTGGTATGCCAGGAGGTATGCCCGGTGGAATGCCAGGTGG<br>AATGCCAGGTGGTATGAATTTCCCAGGAGGTATGCCCGGAGCAGGAATGCCAGGAAATGCCCCAGC<br>TGGAAGTGGACCAACAGTTGAAGAAGTTGATAAGCTTGCGGCCGCACTCGAGCACCACCACCACC<br>ACCACTGA

Supplemental Figures

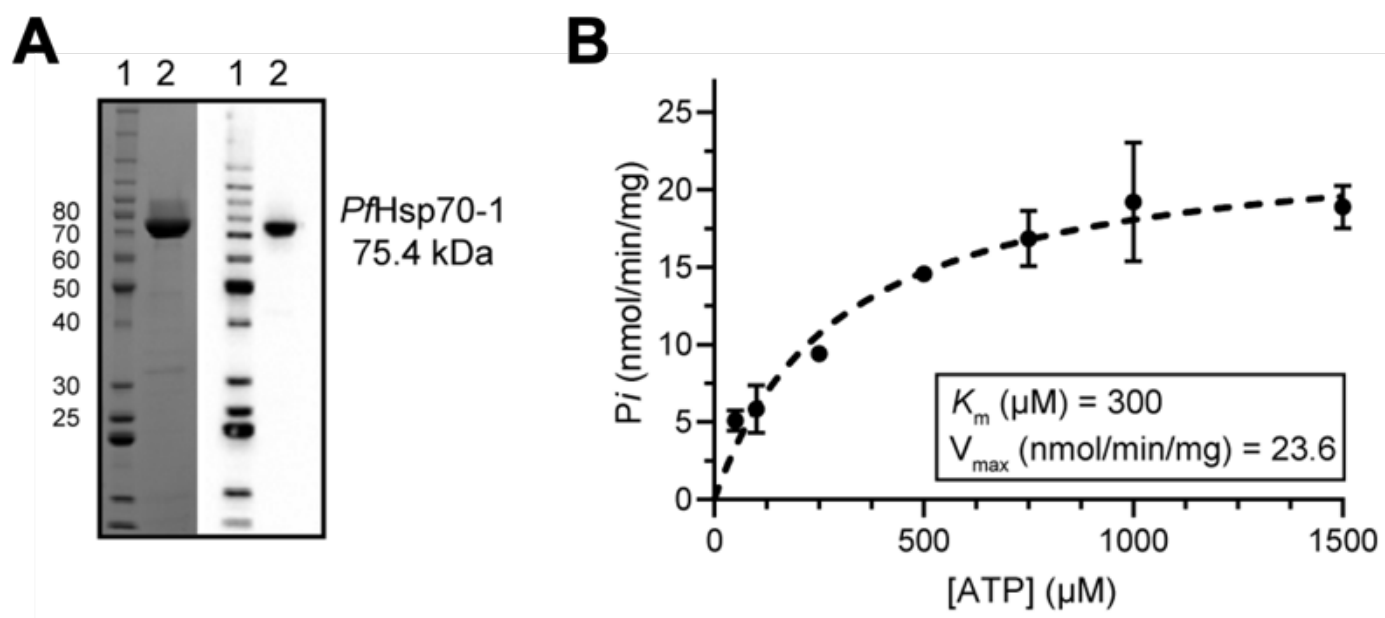

C

Relevant Kinetic Values

Construct	K_m (μM)	V_{max} (nmol/min/mg)	Reference
***Pf*Hsp70-1**	300	23.6	This Study
***Pf*Hsp70-1**	428	24.6	[1]
***Pf*Hsp70-z**	283	19.8	[1]
**HSPA1A**	85	5.5	[2]

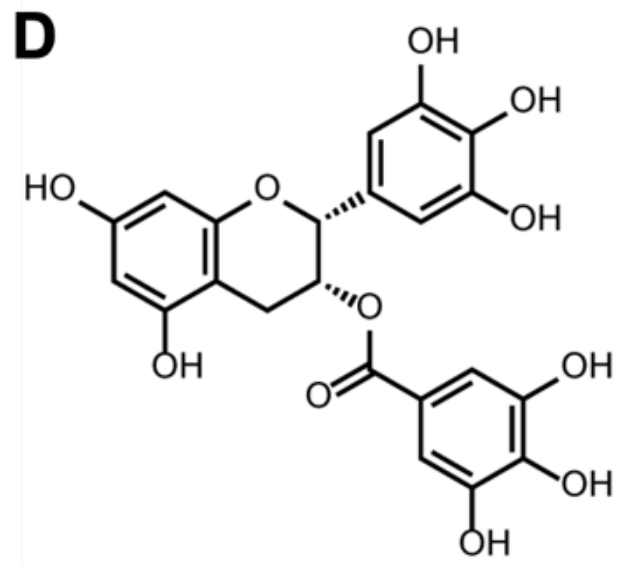

Figure S1. *Pf*Hsp70-1 purification and characterization. (A) SDS-PAGE gel (left panel) and anti-His western blot (right panel) of purified *Pf*Hsp70-1. Lane 1, BenchMark ladder; Lane 2, purified *Pf*Hsp70-1. (B) Basal ATPase activity of purified *Pf*Hsp70-1, data are shown as the average ± SEM (n=3). (C) Kinetic parameters of *Pf*Hsp70-1 from this study compared to previous reports and related Hsp70 protein (1, 2). (D) Structure of non-specific nucleotide binding inhibitor epigallocatechin-3-gallate (EGCG).

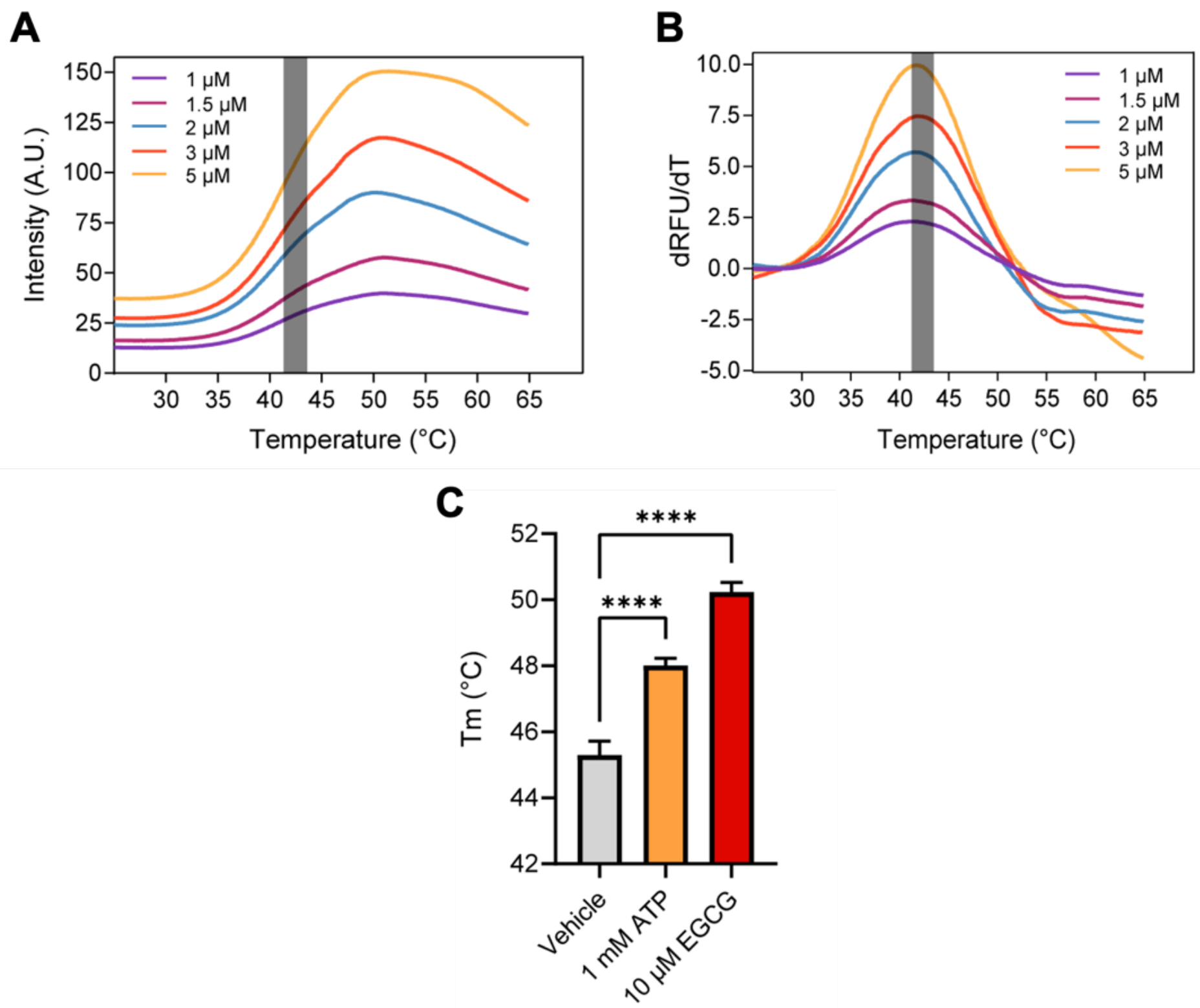

Figure S2. *Pf*Hsp70-1 thermal shift assay optimization. (A) Melting temperature (Tm) of varying purified *Pf*Hsp70-1 concentrations with DMSO, approximate Tm is indicated with grey bar. (B) Tm was calculated by taking the first derivative of the fluorescence values from A with respect to temperature. Tm indicated with grey bar. (C) Tm of 3 μM *Pf*Hsp70-1 with 7% DMSO (grey), 1 mM ATP (orange), 10 μM EGCG (red). Data shown as the average ± SEM (n=3). ****$p < 0.0001$ (unpaired t-test).

NBD SBDβ SBDα, C-term Linker

```
HsHsp70-1      -----------MAKAAAIGIDLGTTYSCVGVFQHGKVEIIANDQGNRTTPSYVAFTDTER      49
PfHsp70-1      MASAKGSKPNLPESNIAIGIDLGTTYSCVGVWRNENVDIIANDQGNRTTPSYVAFTDTER      60
PbHsp70-1      -MANAKAKPNLPESNIAIGIDLGTTYSCVGVWRNENVDIIANDQGNRTTPSYVAFTDTER      59
                            .  ***************::: :*:**********************

HsHsp70-1      LIGDAAKNQVALNPQNTVFDAKRLIGRKFGDPVVQSDMKHWPFQVIND-GDKPKVQVSYK      108
PfHsp70-1      LIGDAAKNQVARNPENTVFDAKRLIGRKFTESSVQSDMKHWPFTVKSGVDEKPMIEVTYQ      120
PbHsp70-1      LIGDAAKNQVARNPENTVFDAKRLIGRKFTESSVQSDMKHWPFTVKSGIEEKPMIEVVYQ      119
               *********** **:************** :  ********** * ..  :** ::* *:

HsHsp70-1      GETKAFYPEEISSMVLTKMKEIAEAYLGYPVTNAVITVPAYFNDSQRQATKDAGVIAGLN      168
PfHsp70-1      GEKKLFHPEEISSMVLQKMKENAEAFLGKSIKNAVITVPAYFNDSQRQATKDAGTIAGLN      180
PbHsp70-1      GEKKLFHPEEISSMVLQKMKENAEAFLGKSIKNAVITVPAYFNDSQRQATKDAGTIAGLN      179
               **.* *:********* **** ***:**  :.*********************.*****

HsHsp70-1      VLRIINEPTAAAIAYGLDRTGKGERNVLIFDLGGGTFDVSILTIDDGIFEVKATAGDTHL      228
PfHsp70-1      VMRIINEPTAAAIAYGLHKKGKGEKNILIFDLGGGTFDVSLLTIEDGIFEVKATAGDTHL      240
PbHsp70-1      VMRIINEPTAAAIAYGLHKKGKGEKNILIFDLGGGTFDVSLLTIEDGIFEVKATAGDTHL      239
               *:***************.:.****:*:*************:***:***************

HsHsp70-1      GGEDFDNRLVNHFVEEFKRKH-KKDISQNKRAVRRLRTACERAKRTLSSSTQASLEIDSL      287
PfHsp70-1      GGEDFDNRLVNFCVEDFKRKNRGKDLSKNSRALRRLRTQCERAKRTLSSSTQATIEIDSL      300
PbHsp70-1      GGEDFDNRLVNFCVEDFKRKNRGKDLSKNSRALRRLRTQCERAKRTLSSSTQATIEIDSL      299
               ***********. **:****:  **:*:*.**:***** **************::*****

HsHsp70-1      FEGIDFYTSITRARFEELCSDLFRSTLEPVEKALRDAKLDKAQIHDLVLVGGSTRIPKVQ      347
PfHsp70-1      FEGIDYSVTVSRARFEELCIDYFRDTLIPVEKVLKDAMMDKKSVHEVVLVGGSTRIPKIQ      360
PbHsp70-1      FEGIDYSVTVSRARFEELCIDYFRDTLIPVEKVLKDAMMDKKSVHEVVLVGGSTRIPKIQ      359
               *****: .:::******** * **.** ****.*:** :**  .:*::***********:*

HsHsp70-1      KLLQDFFNGRDLNKSINPDEAVAYGAAVQAAILMGDKSENVQDLLLLDVAPLSLGLETAG      407
PfHsp70-1      TLIKEFFNGKEACRSINPDEAVAYGAAVQAAILSGDQSNAVQDLLLLDVCSLSLGLETAG      420
PbHsp70-1      TLIKEFFNGKEACRSINPDEAVAYGAAVQAAILSGDQSNAVQDLLLLDVCSLSLGLETAG      419
               .*:::****::  :******************* **:*: *********. *********

HsHsp70-1      GVMTALIKRNSTIPTKQTQIFTTYSDNQPGVLIQVYEGERAMTKDNNLLGRFELSGIPPA      467
PfHsp70-1      GVMTKLIERNTTIPAKKSQIFTTYADNQPGVLIQVYEGERALTKDNNLLGKFHLDGIPPA      480
PbHsp70-1      GVMTKLIERNTTIPAKKSQIFTTYADNQPGVLIQVYEGERALTKDNNLLGKFHLDGIPPA      479
               **** **:**:***:*::******:****************:********:*.*.*****

HsHsp70-1      PRGVPQIEVTFDIDANGILNVTATDKSTGKANKITITNDKGRLSKEEIERMVQEAEKYKA      527
PfHsp70-1      PRKVPQIEVTFDIDANGILNVTAVEKSTGKQNHITITNDKGRLSQDEIDRMVNDAEKYKA      540
PbHsp70-1      PRKVPQIEVTFDIDANGILNVTAVEKSTGKQNHITITNDKGRLSPEEIDRMVNDAEKYKA      539
               ** ******************* .:***** *:*********** :**:***::******

HsHsp70-1      EDEVQRERVSAKNALESYAFNMKSAVEDEGLKGKISEADKKKVLDKCQEVISWLDANTLA      587
PfHsp70-1      EDEENRKRIEARNSLENYCYGVKSSLEDQKIKEKLQPAEIETCMKTITTILEWLEKNQLA      600
PbHsp70-1      EDEENKKRIEARNSLENYCYGVKSSLEDQKIKEKLQPNEVETCMKSVTSILEWLEKNQLA      599
               *** :::*:.*:*:**.*.:.:**::**: :* *:.  : :. :..   ::.**: * **

HsHsp70-1      EKDEFEHKRKELEQVCNPIISGLYQGAGGPGP---GGFGAQGP-----------------      627
PfHsp70-1      GKDEYEAKQKEAESVCAPIMSKIYQDAAGAAGGMPGGM------------PGGMPGGMPG      648
PbHsp70-1      GKDEYEAKQKEAEAVCSPIMSKIYQDAGAAAGGMPGGMPGGMPGGMPGGMPGGMPGGMPG      659
                ***:* *:** * ** **:* :**.*.. .    **:

HsHsp70-1      --------------------KGGSGSGPTIEEVD      641
PfHsp70-1      GMNFPGGMPGA-----GMPGNAPAGSGPTVEEVD      677
PbHsp70-1      GMNFPGGMPGGMPGGMGAPAGAPAGSGPTVEEVD      693
                                    . :*****:****
```

Figure S3. Hsp70 sequence alignment. ClustalW sequence alignment of *Hs*Hsp70-1 (HSPA1A), *Pf*Hsp70-1, and *Pb*Hsp70-1. The predicted N-terminal domain (blue), β-C-terminal domain (green), and α-C-terminal domain (yellow) are highlighted. Linker regions between domains shown in white box. "*" denotes a residue that is identical in all sequences, ":" denotes residue substitutions with strongly similar properties within each sequence, and "." denotes residue substitutions with weakly similar properties. *Pf*Hsp70-1 and *Pb*Hsp70-1 are 94% identical.

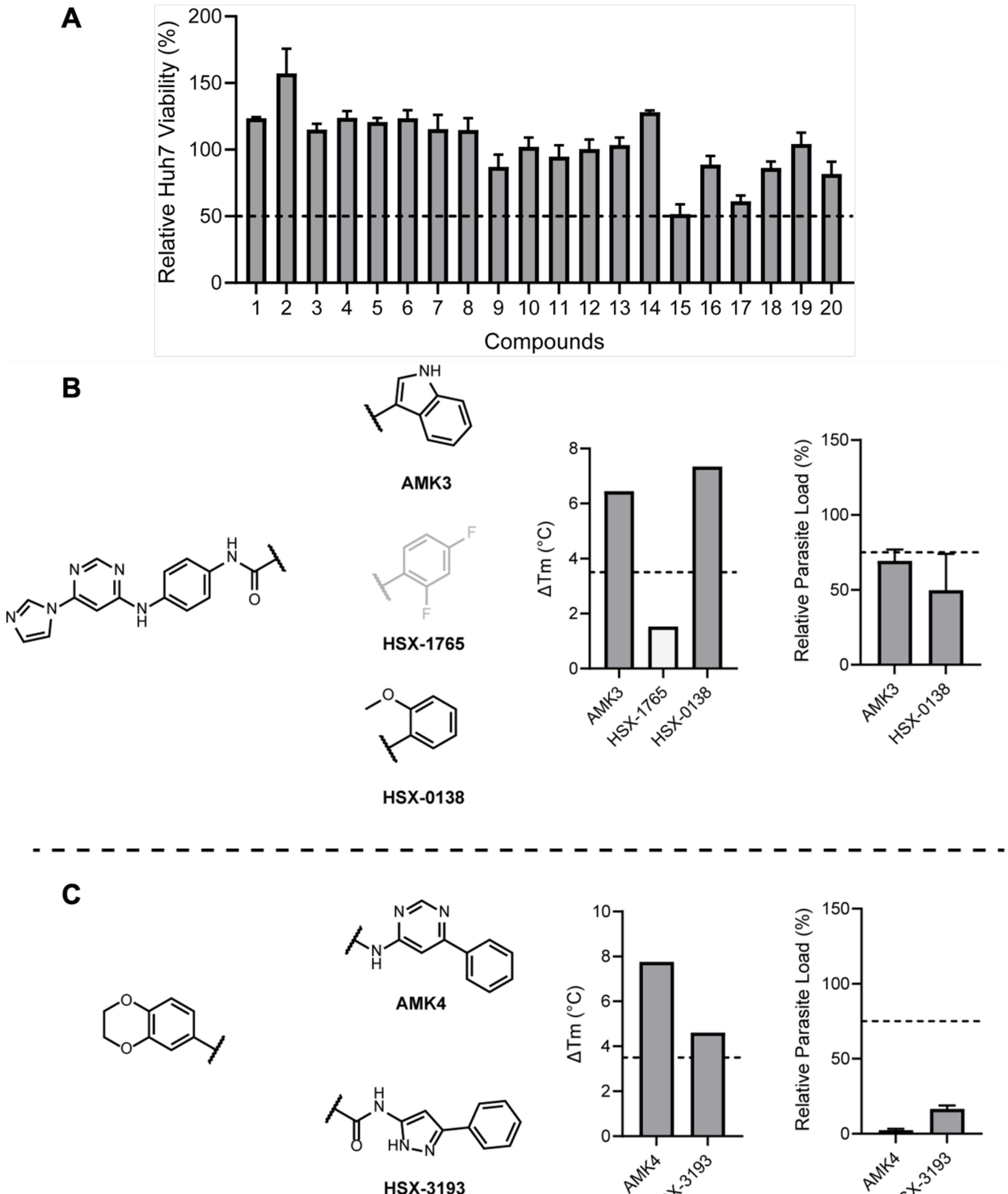

Figure S4. Cell viability of confirmed actives and structural analogs within *Pf*Hsp70-1 screen. (A) Cell viability of confirmed actives (1–20) tested at 10 μM in Huh7 cells. Dotted black line denotes 50% viability. Data are shown as the average ± SEM (n=3). (B) 2 screening actives (in black) contained an imidazole pyrimidine scaffold. **AMK3** (compound **16**) and HSX-0138 shifted the *Pf*Hsp70-1 Tm at 30 μM and inhibited parasite viability at 10 μM, but HSX-1765 (in light grey) did not exhibit a Tm shift. (C) 2 screening actives (in black) contained 1,4-benzodioxane scaffolds. Both **AMK4** (compound **17**) and HSX-3193 shifted the *Pf*Hsp70-1 Tm at 30 μM and inhibited parasite viability at 10 μM. Selection criteria for Tm shift (≥ 3.5 °C) and parasite inhibition (>25%) indicated (black dashed line). Among the analogs **AMK3** and **AMK4** were prioritized for their anti-*Plasmodium* activity and commercial availability.

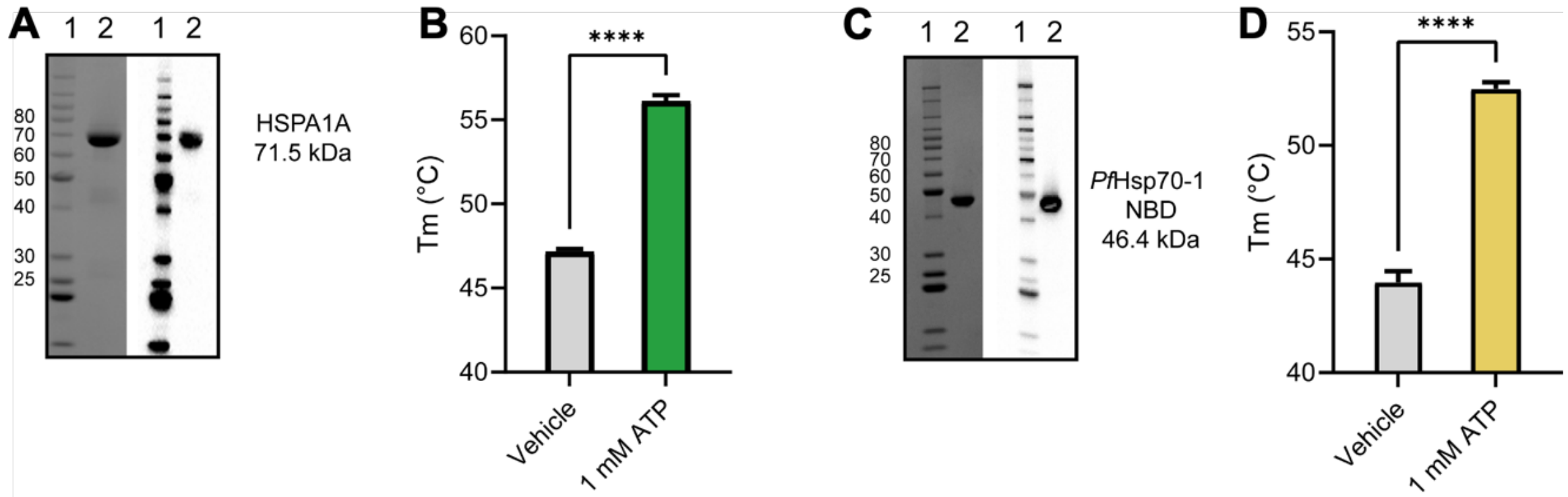

Figure S5. Purification and ATP-binding of HSPA1A and *Pf*Hsp70-1 NBD. (A) SDS-PAGE gel (left panel) and western blot (right panel) of HSPA1A. Lane 1, BenchMark ladder; Lane 2, purified HSPA1A. (B) Tm of 3 µM HSPA1A with ATP. Data shown as the average ± SEM (n=3). ****p < 0.0001 (unpaired t-test). (C) SDS-PAGE gel (left panel) and western blot (right panel) of *Pf*Hsp70-1 NBD. Lane 1, BenchMark ladder; Lane 2, purified *Pf*Hsp70-1 NBD. (D) Tm of 3 µM *Pf*Hsp70-1 NBD with ATP. Data shown as the average ± SEM (n=3). ****p < 0.0001 (unpaired t-test).

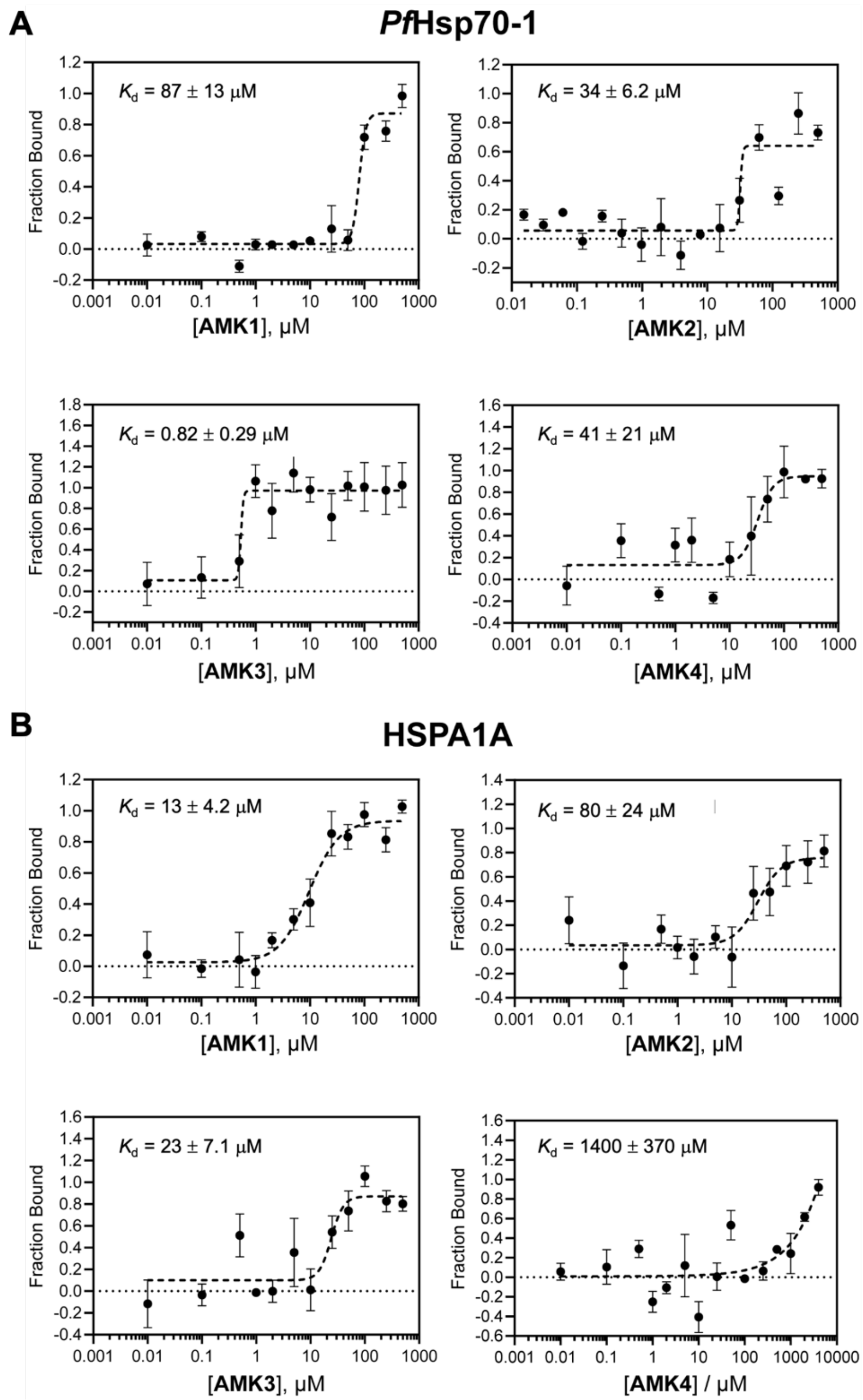

Figure S6. Determination of compound binding to Hsp70-1 by MST. (A) Binding curves for **AMK1–4** to *Pf*Hsp70-1. Data and determined K_d values are shown as the average ± SEM (n≥3). (B) Binding curves for **AMK1–4** to HSPA1A. Data and determined K_d values are shown as the average ± SEM (n≥3). Each reaction contained 20 nM purified RED-NHS labeled Hsp70-1.

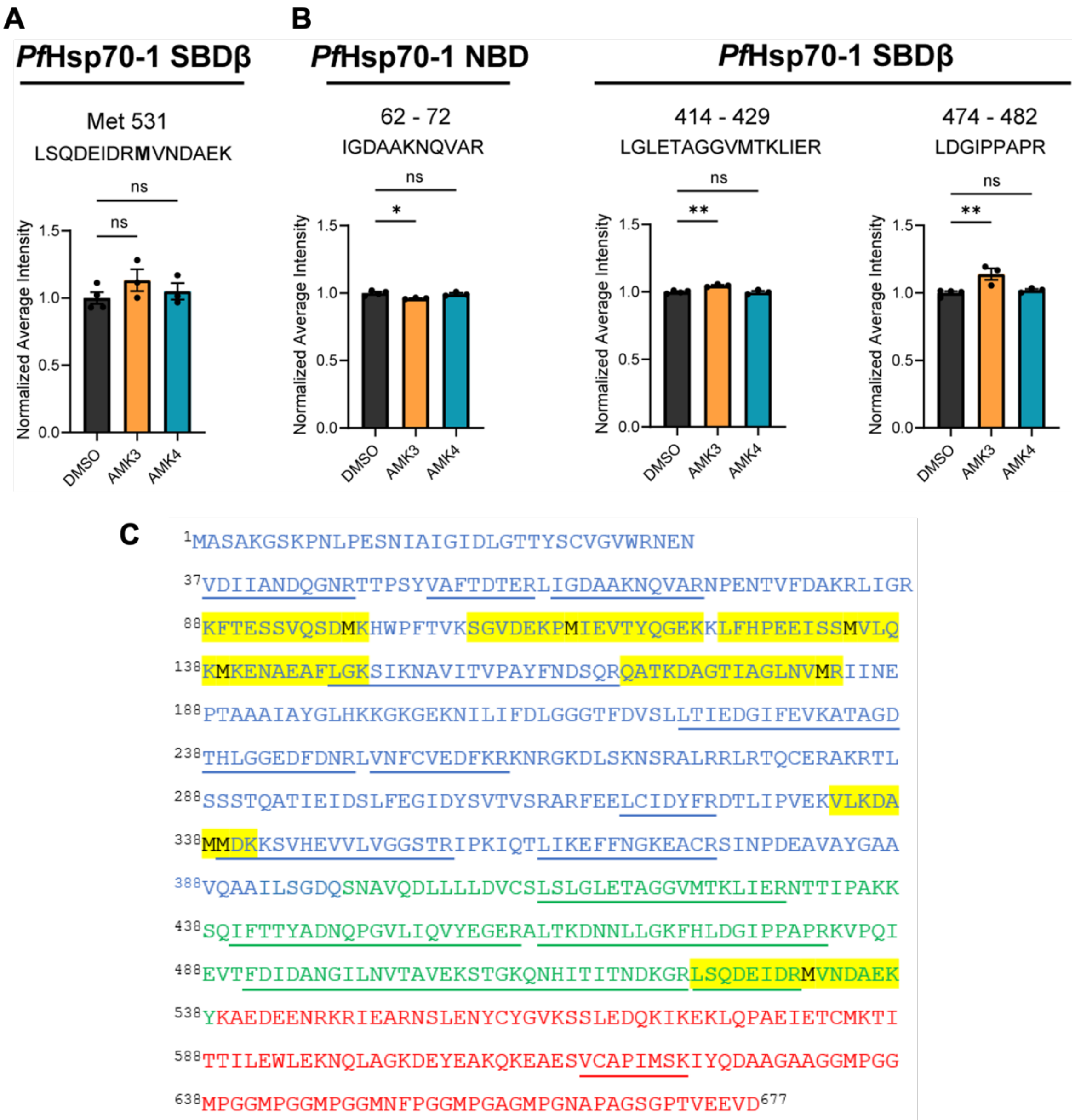

Figure S7. *Pf*Hsp70-1 proteomic analysis of **AMK3** and **AMK4** binding. (A) Oxidized peptide, LSQDEIDRMVNDAEK, from SPROX experiment which maps to the *Pf*Hsp70-1 SBDβ showed no change in stability with 250 μM **AMK3** (orange) or 250 μM **AMK4** (blue) compared to DMSO (grey). Data shown as the average ± SEM (n=3-4). ns: not significant (one-way ANOVA). (B) STEPP-PP performed on *E. coli* over-expressing *Pf*Hsp70-1 lysate incubated with DMSO (grey), 250 μM **AMK3** (blue), or 250 μM **AMK4** (orange). A semitryptic peptide mapping to the *Pf*Hsp70-1 NBD showed significant destabilization with **AMK3** treatment but not with **AMK4** relative to the DMSO control. Two peptides mapping to the *Pf*Hsp70-1 SBDβ showed significant stabilization with **AMK3** compared to DMSO. Data shown as the average ± SEM (n=3-4). **$p < 0.01$; *$p < 0.05$; ns: not significant (one-way ANOVA). (C) Summary of *Pf*Hsp70-1 proteomic coverage. Amino acid sequence is labeled (1-677) with the NBD (blue), SBDβ (green), and SBDα (red) indicated. Peptide coverage observed for SPROX highlighted with oxidized methionines detected shown in black. Semitryptic peptide coverage observed using STEPP-PP are underlined.

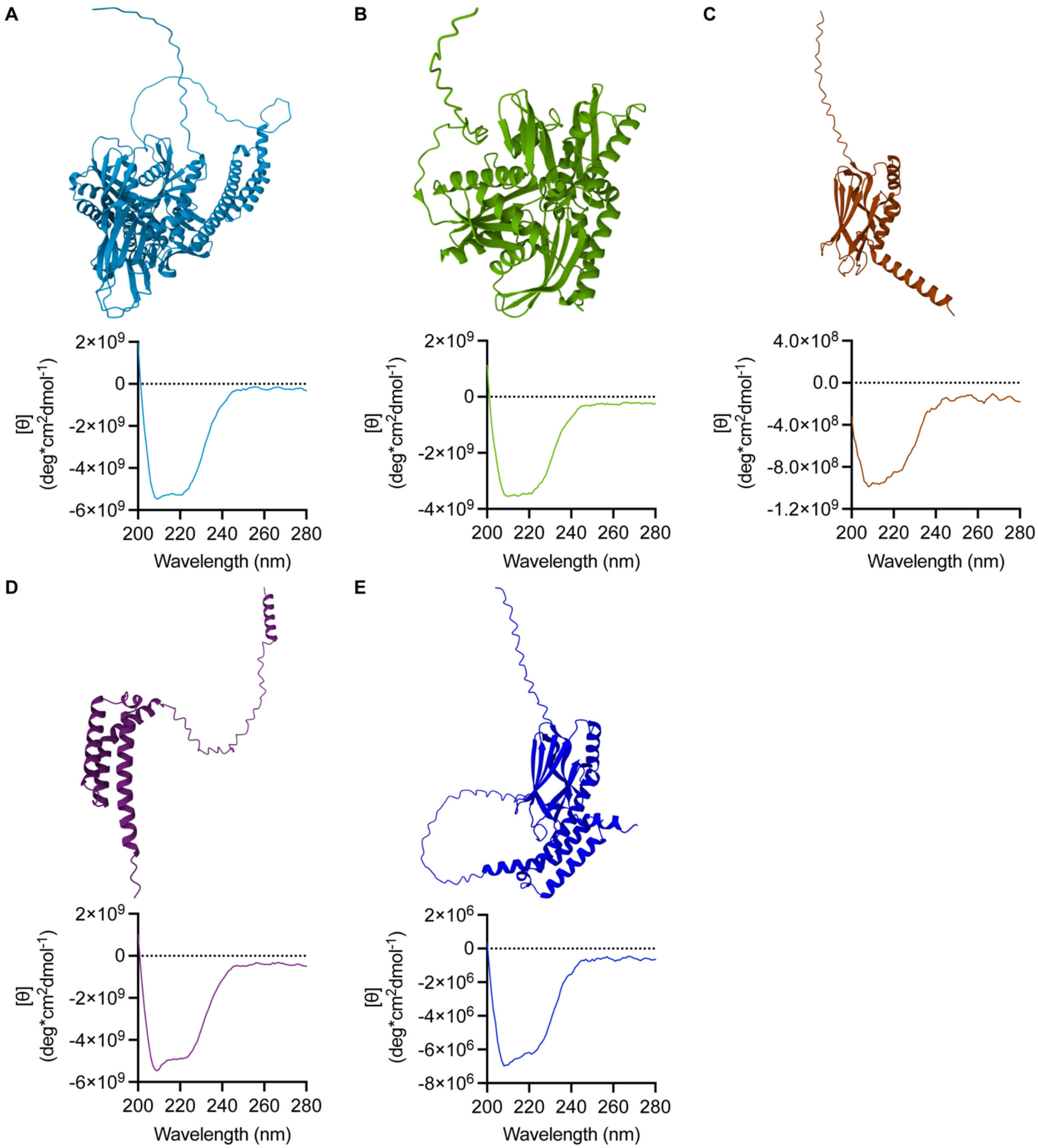

Figure S8. AlphaFold3 homology models and circular dichroism (CD) spectra of purified *Pf*Hsp70-1 constructs. CD spectra of (A) *Pf*Hsp70-1 (75.4 kDa), (B) *Pf*Hsp70-1 NDB (46.4 kDa), (C) *Pf*Hsp70-1 SBDβ (22.4 kDa), (D) *Pf*Hsp70-1 SBDα + DR (16.5 kDa), and (E) *Pf*Hsp70-1 full C-terminal domain (33.0 kDa) were collected and normalized by conversion to molar ellipticity. All spectra are consistent with AlphaFold3 homology models.

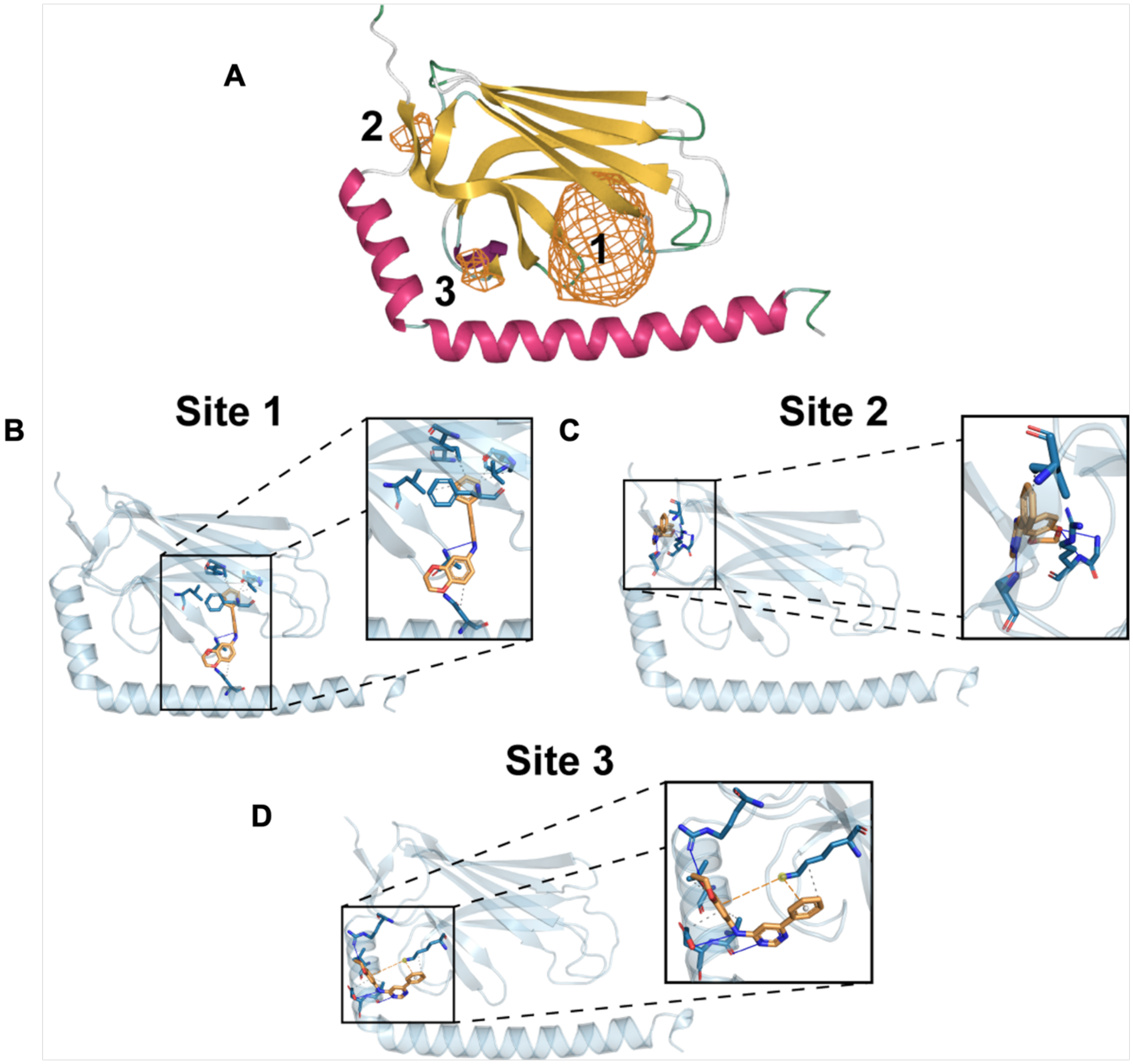

Figure S9. *Pf*Hsp70-1 SBDβ docking interactions. (A) Alphafold2 model of *Pf*Hsp70-1 SBDβ construct (residues 398-572) with potential ligand binding sites (DeepSite) shown as wireframe (orange). Three potential sites annotated. (B) PLIP binding interactions of **AMK4** (tan) docked with AutoDock Vina into Alphafold2 model of *Pf*Hsp70-1 SBDβ in Site 1 (B), Site 2 (C) and Site 3 (D). Interacting residues shown as sticks. Hydrogen bonds denoted by blue lines, hydrophobic interactions as black dashes, and cation – pi interactions shown as connected spheres.

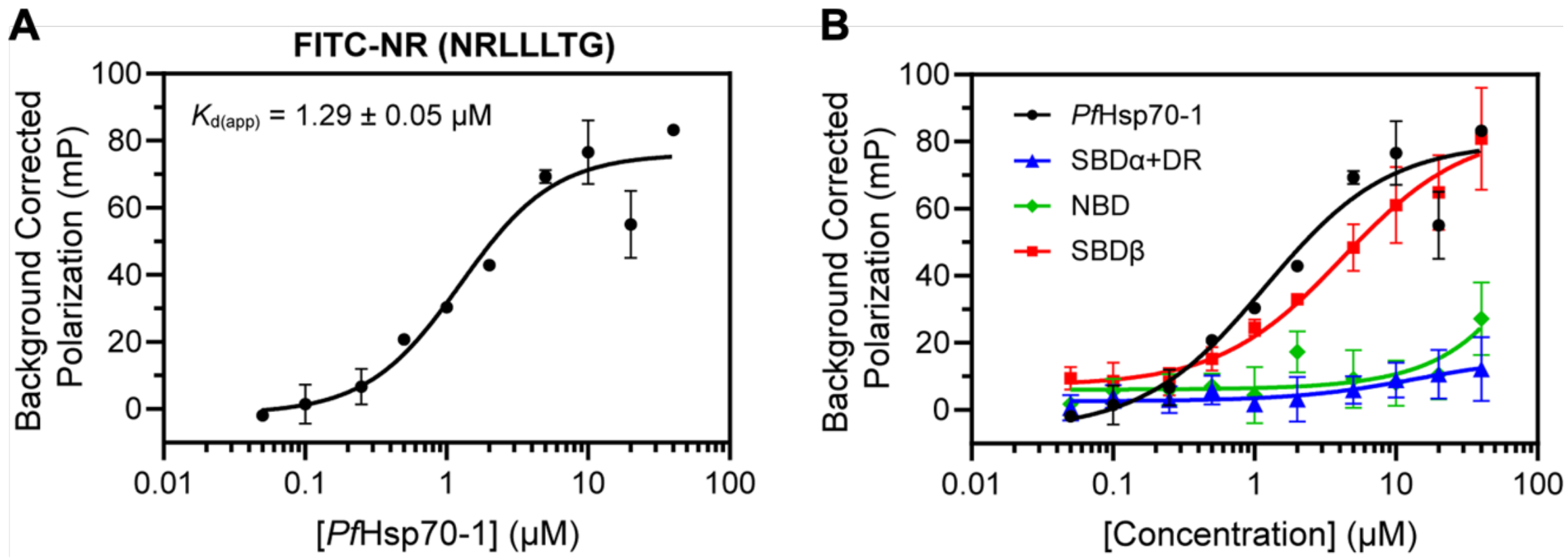

Figure S10. *Pf*Hsp70-1 peptide binding. (A) Serial dilution of *Pf*Hsp70-1 binding to FITC-NR peptide. Data was background corrected to an unbound tracer control lacking *Pf*Hsp70-1. Data shown as the average ± SEM (n≥3) and fit to non-linear curve. NR peptide sequence shown above plot and calculated apparent K_d displayed as average ± SEM. (B) Normalized fluorescence polarization of purified full-length *Pf*Hsp70-1 (black), NBD (green), SBDβ (red), and SBDα with disordered region (DR) (blue). Data was background corrected to an unbound tracer control without protein. Data shown as the average ± SEM (n=5).

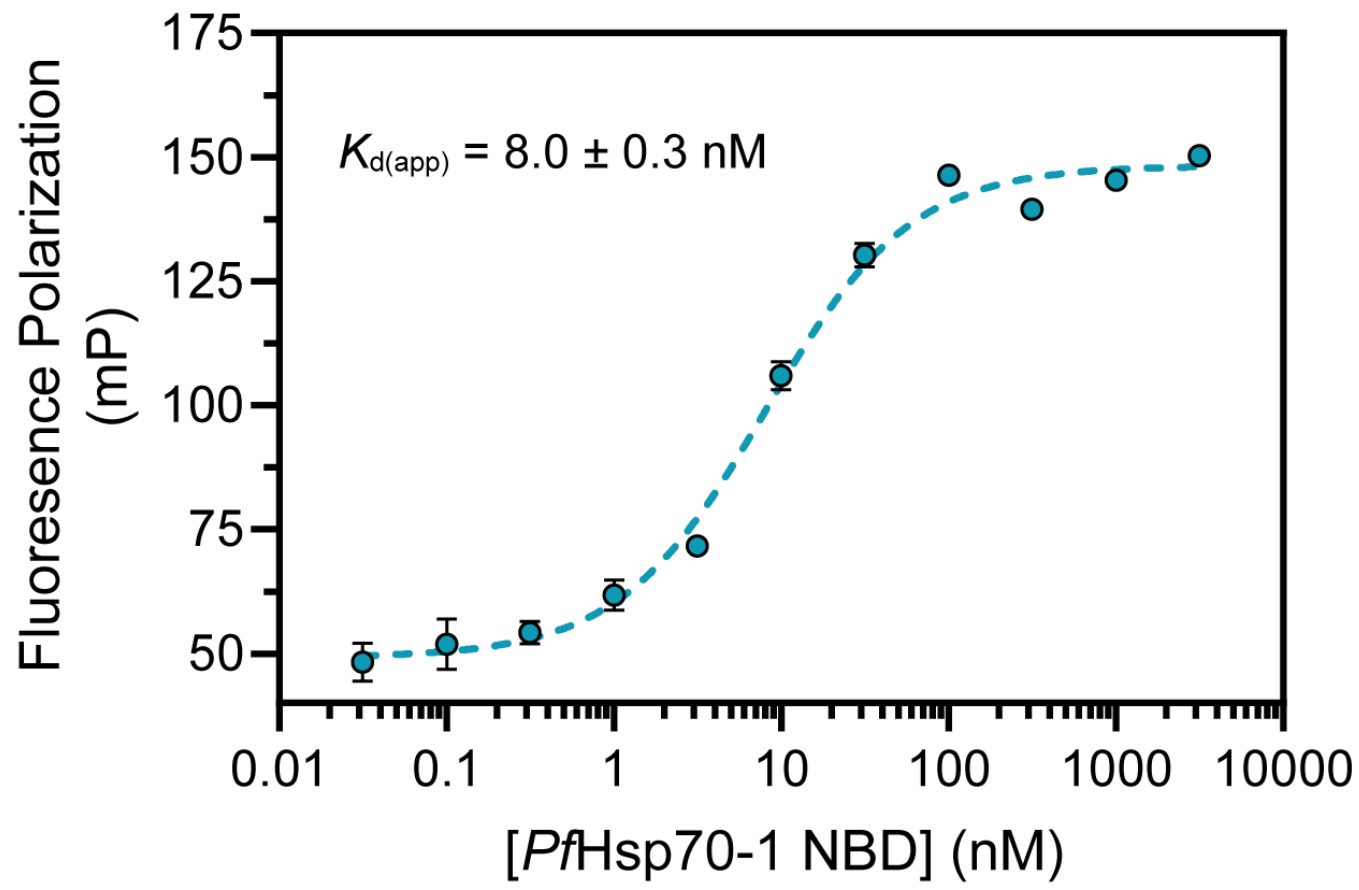

Figure S11. Fluorescein-12-ATP (F12-ATP) binding to *Pf*Hsp70-1 NBD. Serial dilution of *Pf*Hsp70-1 NBD binding to F12-ATP. Data shown as the average ± SEM (n=3) and fit to non-linear curve. Calculated apparent K_d displayed as average ± SEM.

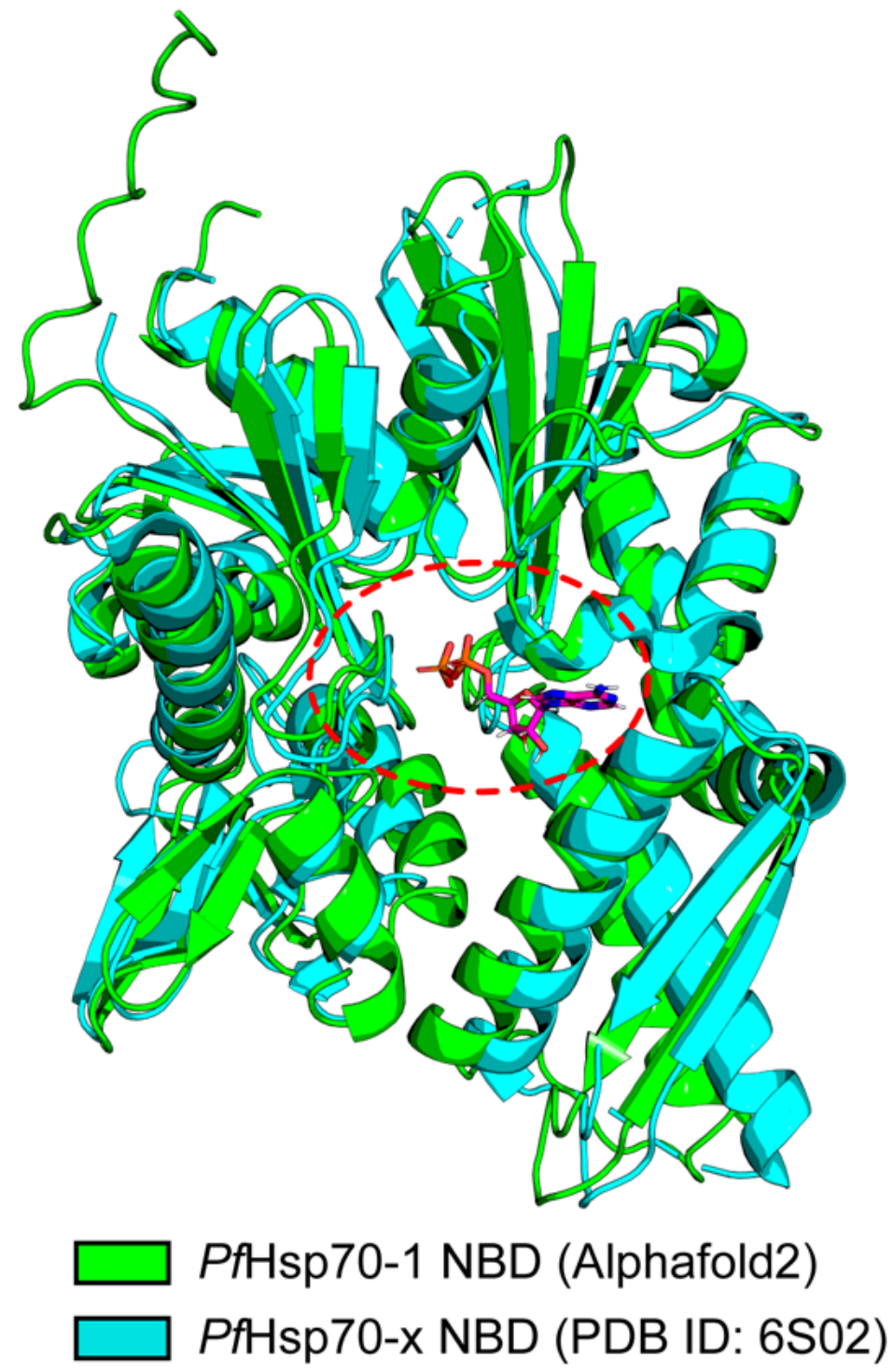

Figure S12. *Pf*Hsp70-1 structure validation for modeling. *Pf*Hsp70-1 NBD (Alphafold2 model, green) and *Pf*Hsp70-x NBD (PDB ID: 6S02, cyan) depicted as cartoons. ADP from PDB ID: 6S02 shown as sticks (purple). Approximate active site shown in red circle.

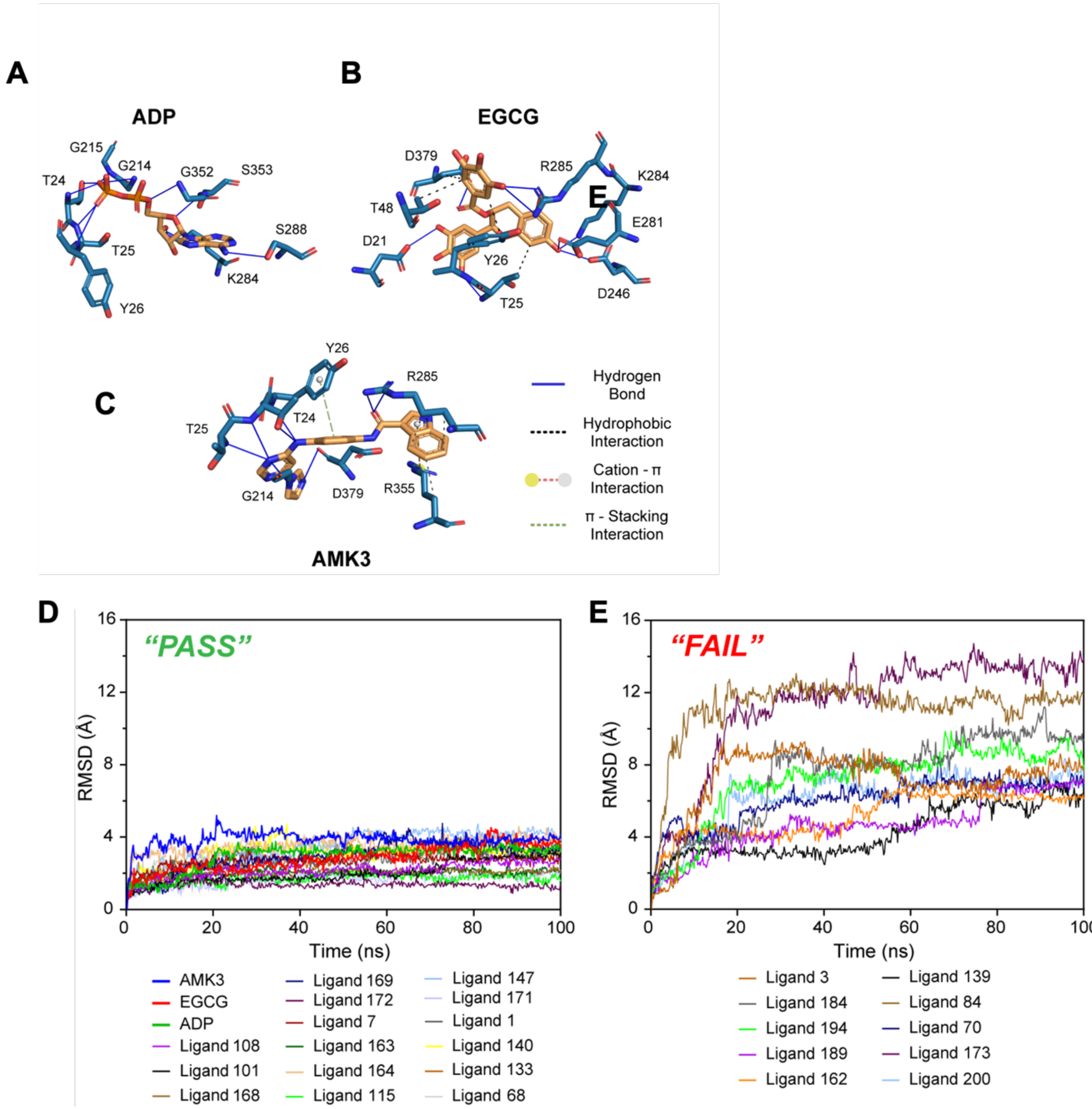

Figure S13. *Pf*Hsp70-1 NBD docking and molecular dynamics. PLIP binding interactions of ADP (A), EGCG (B) and **AMK3** (C) docked by AutoDock Vina into *Pf*Hsp70-1 NBD (Alphafold2 model). Molecules shown in tan and interacting residues shown as sticks and labeled. Hydrogen bonds (blue lines), hydrophobic interactions (black dashes), cation – pi interactions (connected spheres), and pi – stacking interactions (green dashes) shown. (D) Ligand RMSD plot of complexes that "pass" visual inspection and average RMSD cutoffs, positive controls (**AMK3**, EGCG, and ADP) shown in bold. 15 of 25 ligands passed this analysis. (E) Ligand RMSD plot of complexes that "fail" visual inspection and average RMSD cutoffs. 10 of 25 ligands failed this analysis. For both, RMSD values were calculated over the entire simulation. Data shown as the average of 2 independent 100 ns replicates.

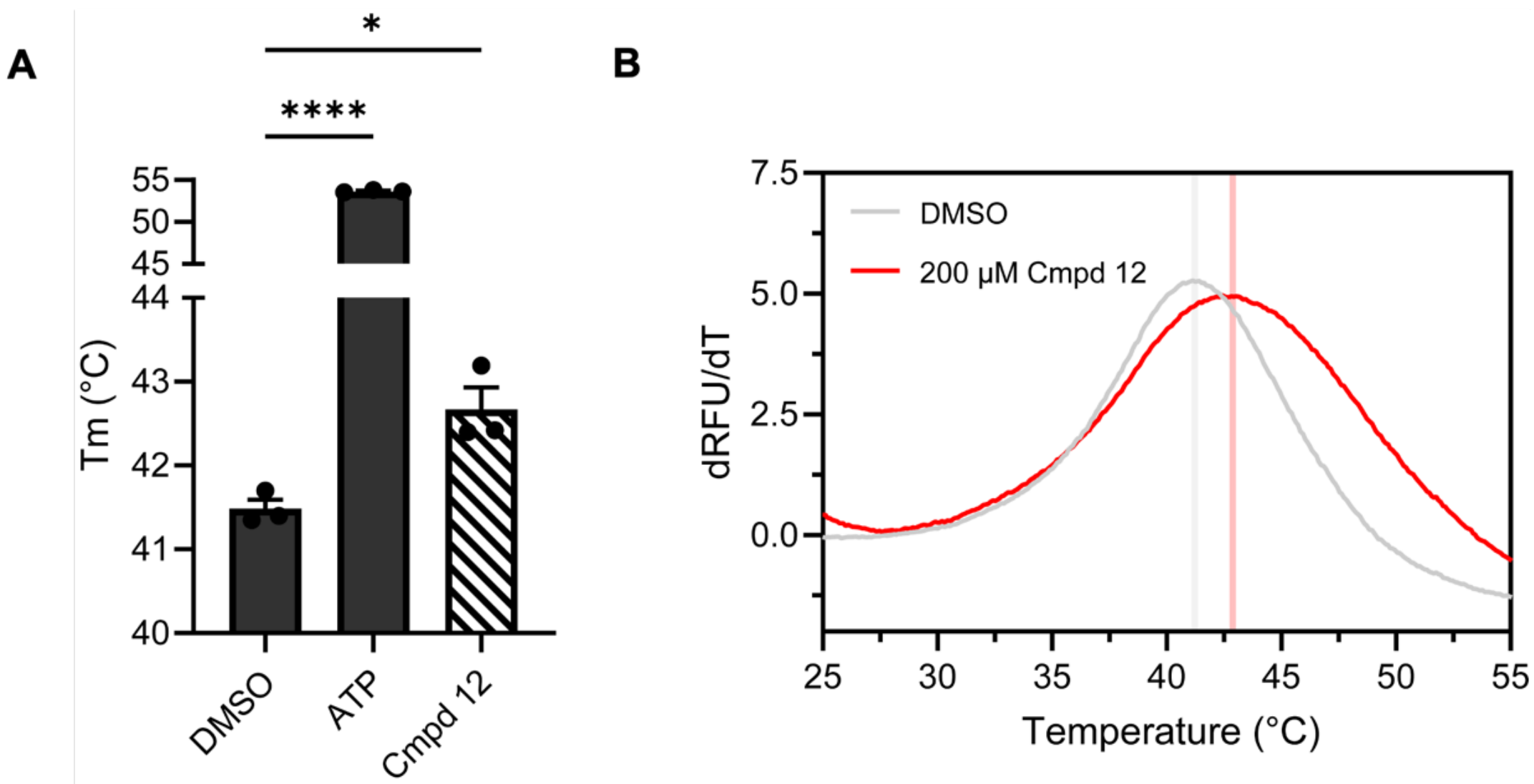

C

	K_d (µM)	
Compound	*Pf*Hsp70-1	HSPA1A
AMK3	0.82 ± 0.29	22.5 ± 7.1
12	0.86 ± 0.35	3.91 ± 2.66

Figure S14. Compound **12** binding to Hsp70-1 by thermal shift and MST. (A) *Pf*Hsp70-1 NBD Tm with ATP and compound **12**. Data shown as the average ± SEM (n=3). ****p < 0.0001; *p < 0.05 (unpaired t-test). (B) Representative derivative data used to calculate the Tm for each condition with Tms indicated by vertical lines. (C) Binding of **AMK3** and compound **12** determined by MST for *Pf*Hsp70-1 and HSPA1A. Data shown as the average ± SEM (n≥3).

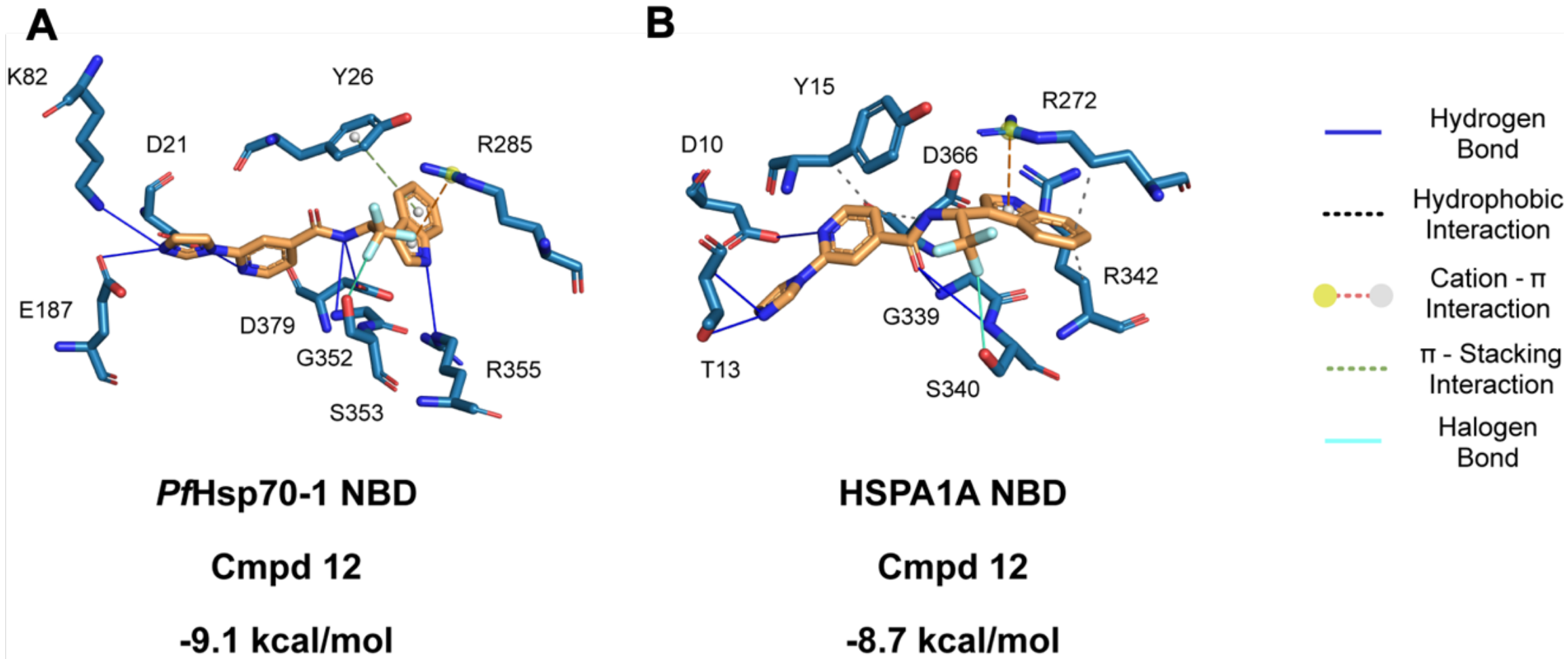

Figure S15. Docking of compound **12** to Hsp70-1. (A) Top position of Compound **12** docked into an *Pf*Hsp70-1 NBD (Alphafold2 model) using AutoDock Vina with high exhaustiveness. The PLIP binding interactions of **12** (tan) with *Pf*Hsp70-1 NBD residues (blue, sticks) are labeled. The calculated AutoDock Vina docking energy shown below the structure. (B) Top position of Compound **12** docked into HSPA1A NBD (PDB ID: 3ATV) using AutoDock Vina with high exhaustiveness. The PLIP binding interactions of **12** (tan) with HSPA1A NBD residues (blue, sticks) are labeled. The calculated AutoDock Vina docking energy shown below the structure. For all docking models, hydrogen bonds (blue lines), hydrophobic interactions (black dashes), cation – pi interactions (connected spheres), pi – stacking interactions (green dashes), and halogen bonds (cyan lines) are shown.

Supplemental References

1. T. Zininga, *et al.*, Overexpression, Purification and Characterisation of the Plasmodium falciparum Hsp70-z (PfHsp70-z) Protein. *PLoS One* **10**, e0129445 (2015).
2. C. L. Olson, *et al.*, Molecular and biochemical comparison of the 70-kDa heat shock proteins of Trypanosoma cruzi. *J. Biol. Chem.* **269**, 3868–3874 (1994).
